# What Is the Nature of Supramolecular Bonding? Comprehensive NBO/NRT Picture of Halogen and Pnicogen Bonding in RPH_2_···IF/FI Complexes (R = CH_3_, OH, CF_3_, CN, NO_2_)

**DOI:** 10.3390/molecules24112090

**Published:** 2019-05-31

**Authors:** Yinchun Jiao, Frank Weinhold

**Affiliations:** 1 Key Laboratory of Theoretical Organic Chemistry and Functional Molecules, Ministry of Education, School of Chemistry and Chemical Engineering, Hunan University of Science and Technology, Xiangtan 411201, China; jiao28@wisc.edu; 2 Theoretical Chemistry Institute and Department of Chemistry, University of Wisconsin-Madison, Madison, WI 53706, USA

**Keywords:** supramolecular bonding, halogen bond, pnicogen bond, hydrogen bond, natural bond orbital, natural resonance theory, fractional bond order, resonance theory

## Abstract

We employ a variety of natural bond orbital (NBO) and natural resonance theory (NRT) tools to comprehensively investigate the nature of halogen and pnicogen bonding interactions in RPH_2_···IF/FI binary complexes (R = CH_3_, OH, CF_3_, CN, and NO_2_) and the tuning effects of R-substituents. Though such interactions are commonly attributed to “sigma-hole”-type electrostatic effects, we show that they exhibit profound similarities and analogies to the resonance-type 3-center, 4-electron (3c/4e) donor-acceptor interactions of hydrogen bonding, where classical-type “electrostatics” are known to play only a secondary modulating role. The general 3c/4e resonance perspective corresponds to a continuous range of interatomic A···B bond orders (*b*_AB_), spanning both the stronger “covalent” interactions of the molecular domain (say, *b*_AB_ ≥ ½) and the weaker interactions (*b*_AB_ ˂ ½, often misleadingly termed “noncovalent”) that underlie supramolecular complexation phenomena. We show how a unified NBO/NRT-based description of hydrogen, halogen, pnicogen, and related bonding yields an improved predictive utility and intuitive understanding of empirical trends in binding energies, structural geometry, and other measurable properties that are expected to be manifested in all such supramolecular interaction phenomena.

## 1. Introduction

Recent computational [1,2,3,4] and experimental [5,6,7,8] studies have called attention to the multiplicity of halogen [9,10,11,12,13], chalcogen [14,15], pnicogen [16], and tetrel bonds [17,18,19] (corresponding to Groups 17, 16, 15, and 14, respectively) that appear necessary to supplement the familiar hydrogen bonds of intermolecular interaction theory [20]. Such proliferating “bond” forms naturally reignite age-old controversies concerning the relative importance of “covalent” versus “electrostatic” contributions in the general theory of intermolecular forces [21,22,23,24]. Of course, all protagonists concur that the only relevant potential terms in the non-relativistic Hamiltonian are of Coulombic form (hence, tautologically “electrostatic” in nature). However, the deeper issue is whether supramolecular complexation (like molecule formation) is primarily governed by exchange-type (exponential) interactions of the short-range quantal regime or by the exchange-free (power–law) behavior of the long-range classical limit. In short, we ask whether orbital-based (quantal) or “dipole–dipole”-type (classical) conceptions provide the more useful starting point for describing intermolecular forces. For present purposes, the former is exemplified by a natural bond orbital [25] (NBO)-based description, and the latter by is exemplified by symmetry adapted perturbation theory (SAPT) [26,27], electrostatic potential models (e.g., of CHELP [28,29] or “sigma hole” [30,31,32] types), or related classical force field [33,34] models.

In the case of hydrogen bonding, the primacy of short-range covalency forces is now widely recognized [20,35,36,37,38]. In the NBO framework [39], a general B:···H—A hydrogen bond is identified with the strength of a *n*_B_→σ*_AH_ donor–acceptor interaction between the lone pair (*n*_B_) of the Lewis base B and the valence antibond (σ*_AH_) of the Lewis acid A—H. In natural resonance theory (NRT) [40,41,42,43] terms, such a *n*_B_→σ*_AH_ interaction represents resonance mixing between the parent natural Lewis structure (NLS) **I** and the secondary “charge–transfer” resonance structure **II** (with respective NRT weightings *w***_I_** and *w***_II_**):

B:···H—A ↔ B(+)—H···:A(−)   **I**        **II**    (1a)

in which the “covalent” (—) and “hydrogen” bond (···) symbols are interchanged [44]. As recognized by Coulson [45], the resonance hybrid (1a) is also the resonance-theoretic equivalent of the Pimentel–Rundle 3-center, 4-electron (3c/4e) molecular obital (MO) formulation of hypervalency [46,47]. In the simple two-resonance limit, the formal *b*_AH_, *b*_BH_ bond orders are necessarily related by the characteristic “bond conservation” relationship [48],

*b*_AH_ + *b*_BH_ = 1 (1b)

which merely reflects the resonance-averaging condition (*w***_I_** + *w***_II_** = 1) for the composite resonance hybrid. Even in the absence of NRT bond order descriptors, the importance of NBO *n*_B_→σ*_AH_ delocalization can be assessed by 2^nd^-order perturbative energy (Δ*E*^(2)^_nσ*_), intermolecular charge-transfer (*Q*_nσ*_), or the deletion of this NBO interaction (Δ*E*^$DEL^_nσ*_), as well as the variational recalculation of structure, energetics, and vibrational frequencies [49]. In contrast to the superficial “on-off” picture of H-bonding that is sometimes suggested by graphical software or inflexible analysis methods, the NBO/NRT descriptors all reflect the continuously variable character of resonance-type bonding, as quantified by fractional bond orders ranging over integer and sub-integer values. All such characteristic NBO/NRT signatures are found in all known H-bonded species, including the paradoxical “anti-electrostatic” H-bonds between like-charged ions [50] that most clearly demonstrate the relatively secondary influence of “electrostatic” modulation on the authentic H-bonding phenomenon.

In the present work, we take up the analogous covalency versus electrostatics questions for title complexes RPH_2_···IF/FI (R = CH_3_, OH, CF_3_, CN, NO_2_) that exhibit a wide range of binding strengths. A smaller subset of such species was previously examined [51] with an NBO/NRT analysis of MP2/aug-cc-pVTZ level computations. The present study extends the choice of species and the variety of NBO/NRT analysis tools to specifically address the broader questions of covalency versus electrostatics in halogen and pnicogen bonding, as well as the general relationships to H-bonding and other types of “X-ogen bonding” that signal a grand unified picture of all such donor-acceptor phenomena. In each case, we exhibit the mutually consistent correlations between NBO/NRT-based descriptors and experimentally measurable properties that illustrate the interpretative and predictive advantages of the unified NBO/NRT conceptual framework compared to alternative analysis methodologies [25].

Note that we ignore the weaker effects of the London dispersion interaction [52], a pure correlation effect that (unlike the *n*-σ* interaction) is strictly absent in uncorrelated Hartree–Fock theory. Though an empirical dispersion correction [53] is often found to improve the performance of density functional theory (DFT) calculations, such correction is only an additive term in the total energy expression, with no direct effect on Kohn–Sham orbitals, electron density, or other properties. Effects of this correction are therefore “invisible” to NBO/NRT analysis at any specific geometry, but, in principle, they can lead to shifts on the potential energy surface at which an analysis is performed. We evaluated the B3LYP-D3 properties for CH_3_PH_2_···IF (see Supplementary Materials for full details) and found that the changes are relatively negligible in magnitude (smaller, e.g., than variations among alternative basis levels discussed below) as well as opposite in sign to that expected for the authentic London dispersion attraction (acting instead to reduce Δ*E*_bind_). Such “corrections” are not considered further in the present work. 

In the following, we first describe the species, computational levels, and NBO/NRT methods to be employed. The latter include NBO Δ*E*^(2)^_nσ*_, charge–transfer *Q*_nσ*_, and $DEL-deletion Δ*E*^$DEL^_nσ*_ measures of donor–acceptor attraction [49], the corresponding pairwise steric-exchange opposition (Δ*E*^(pw)^_nσ_) of filled orbitals [54] that completes the 3c/4e description of overall bonding energetics, and the various NRT-based regularities and correlations with measurable properties that are considered diagnostic of H-bonding [22]. We then document the systematic application of these NBO/NRT methods to the title species to exhibit their mutual coherence and consistency with well-established features of H-bonding. The concluding summary emphasizes how a balanced NBO/NRT description of leading donor–acceptor interactions can lead to a unified conceptual picture of supramolecular bonding that encompasses the entire range of chemically significant (“non-innocent”) complexation and ligation phenomena. 

## 2. Computational Methods and Results

Our studies began with a larger data set of dihalogen complexes RPH_2_···XY that included all possible (X, Y = F, Cl, Br, I) dihalogen species of the first four periodic rows. However, from a qualitative conceptual perspective, it soon became apparent that the properties of such complexes vary in a smooth and chemically reasonable fashion with dihalogen polarity, as maximized in the iodine–fluorine (IF) species. Accordingly, we focus here on the “polar extremes” of RPH_2_···IF versus RPH_2_···FI complexation, corresponding to opposed signs of any envisioned “dipole–dipole” contributions to intermolecular binding. A direct comparison of these two extremes thereby allows one to recognize the important modulating influence of classical dipole–dipole forces (i.e., in strengthening or weakening the underlying quantal interactions), while also verifying that resonance-type covalency forces yield robust supramolecular bonding even when the presumed classical electrostatic prerequisites for such complexation are profoundly violated. While the computational results presented here focus only on the extremal RPH_2_···IF versus RPH_2_···FI limits of this broader picture, the Supplementary Information (SI) includes results for all remaining dihalogen species to illustrate the generality of the resonance-type 3c/4e interaction picture to be sketched below.

Though the conceptual picture we describe is insensitive to many details of the chosen computational method and basis set [55], the inclusion of iodine (for which common Pople-type 6-311++G** basis sets are unavailable) mandates the adoption of a relativistically corrected basis set (e.g., of the pseudopotential LANL2DZ type). For present qualitative purposes, we therefore adopt an unconventional but simple “mixed-PP” basis set of 6-311++G** for all RPH_2_ monomers and LANL2DZ for dihalogen monomers of each complex. We employ simple B3LYP density functional methodology throughout this work, but alternative MP2 evaluations at different basis levels were also performed for comparison (see SI). All structures were optimized, and frequencies were computed to confirm equilibrium geometries corresponding to minimum energy points. The binding energy, Δ*E*_bind_ = *E*_(AB)_ − *E*_(A)_ − *E*_(B)_, was calculated as the direct energy difference between optimized dimer and relaxed monomers. All electronic structure calculations were performed with the Gaussian 16 program package [56]. NBO/NRT analyses were performed with *NBO 7.0* software [57], and both NBO/NRT analyses and orbital imagery were obtained with the *NBOPro@Jmol* program [58].

Table 1 summarizes the key structural and binding energy parameters of RPH_2_···IF and RPH_2_···FI species as obtained at the adopted “B3LYP/mixed-PP” level, showing the wide range of tuning by various R-substituents. Despite the fact that all complexes are appreciably bound (by circa 3–15 kcal/mol, in defiance of superficial “dipole–dipole” or Coulombic point–charge expectations), interesting structural contrasts are immediately evident between these two classes of species. Focusing first on the orientation angles (Θ) in Table 1, one can see that the RPH_2_···IF species all maintain nearly linear P···IF alignment (Θ_PIF_ ≈ 180°) and a circa 120° bend angle between RP and IF axes (Θ_RPI_ ≈ 120°). In contrast, the RPH_2_···FI species are noticeably tilted away from P···FI linearity (Θ_PFI_ ≈ 160°), instead adopting increasingly near-linear alignment with respect to RP and FI axes (Θ_RPF_ ≈ 170°). Such structural tendencies suggest that the principal underlying “linearizing” (H-bond-like) interaction differs in the two cases and that other RPH_2_···XY dihalogen species of intermediate polarity will likewise exhibit intermediate orientational preferences between these two competing tendencies. Indeed, this is the broader picture obtained from the full set (X, Y = F, Cl, Br, I) of optimized dihalogen structures, with the homopolar RPH_2_···X_2_ species forming the approximate “dividing line” between RPH_2_···IF-like versus RPH_2_···FI-like structural propensity. These structural features of RPH_2_···IF-like (“P···IF”) versus RPH_2_···FI-like (“RP···F”) complexes are also consistently exhibited at other theory levels, as shown in SI Tables S1–S3. All such comparisons indicate the adequacy of the adopted B3LYP/mixed-PP model and the extremal RPH_2_···IF versus RPH_2_···FI species for describing the full range of RPH_2_···XY complexation (X, Y = F, Cl, Br, I; R = CH_3_, OH, CF_3_, CN, NO_2_), including general consistency with previous MP2/aug-cc-pVTZ-level calculations.

**Figure 1:**
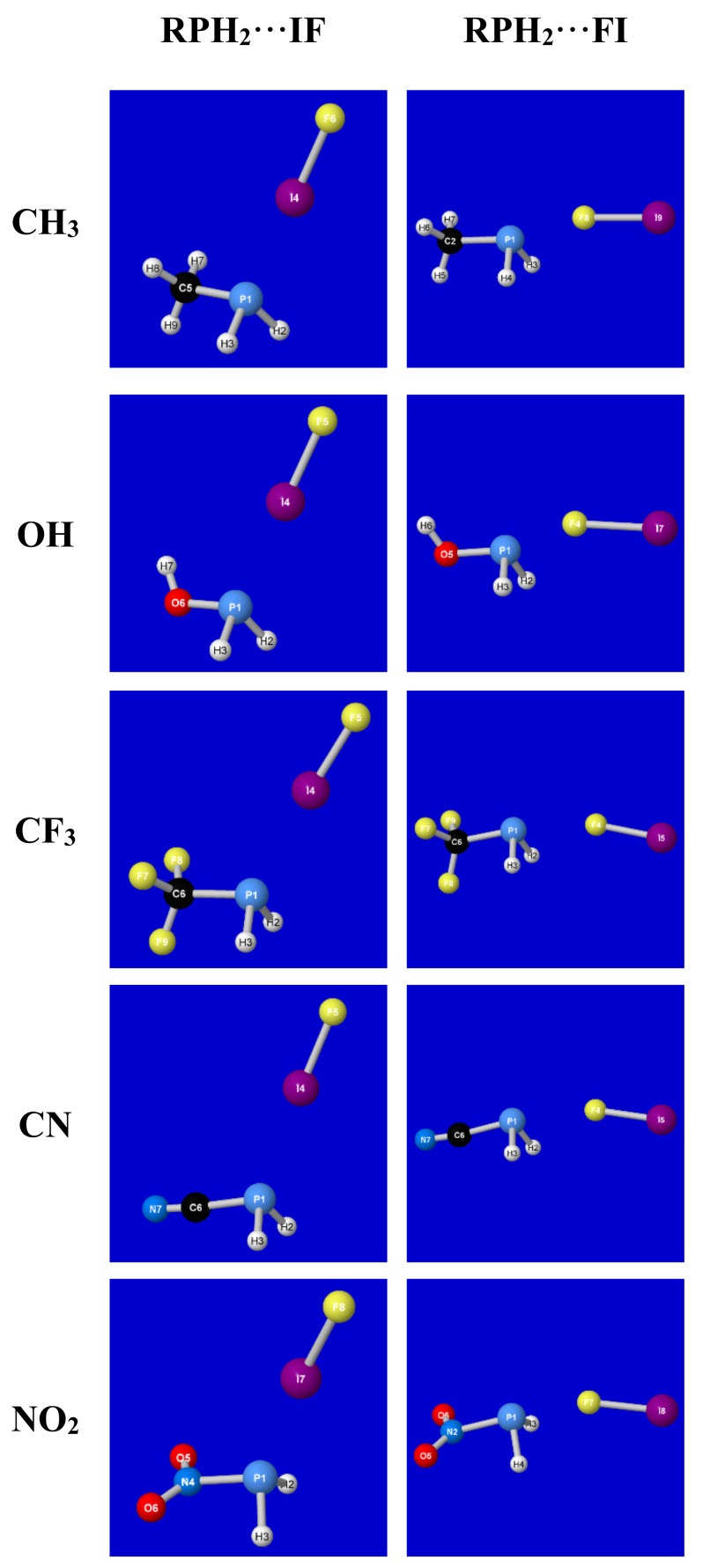
Optimized structures of RPH_2_···IF and RPH_2_···FI complexes (cf. Table 1).

Figure 1 depicts the numerical structural data of Table 1 in graphical form, showing the distinct structural motifs that characterize each extremal “type” of association. Though the graphical imagery primarily allows for the visualization of the angular features of each complex, the progressive shortening of phosphorus–halogen bond length *R*_P···F_ or *R*_P···I_ (as well as concomitant elongation of dihalogen bond length *R*_IF_) with increasing binding energy Δ*E*_bind_ is also discernable in the details of the figure panels. From both Table 1 and Figure 1, one can recognize that the structural effects of different R-substituents are modest compared to that of reversing dihalogen polarity. 

**Figure 2:**
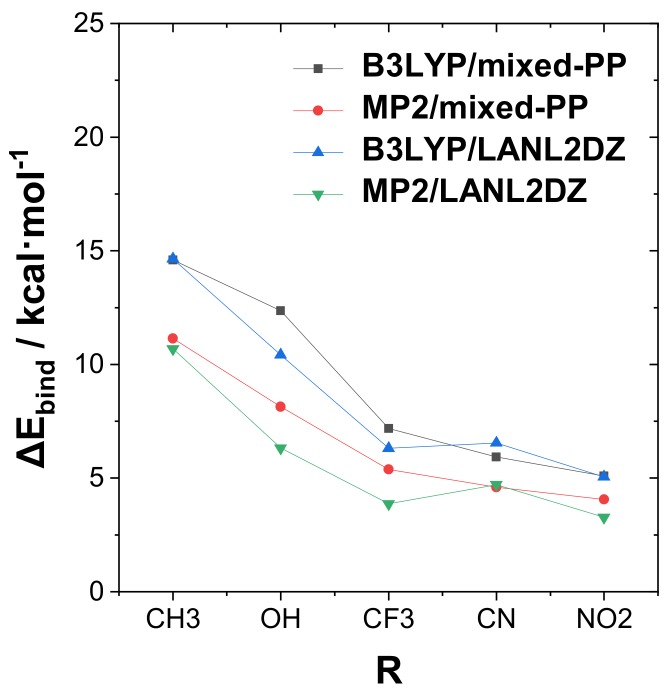
Calculated trend of Δ*E*_bind_ with substituent R in the RPH_2_···IF complexes for four theoretical levels. Note the reversed ordering of the cyano substituent effect on binding energy when diffuse functions are absent (“pure” LANL2DZ; see text).

Figure 2 presents a graphical display of the trends in tuning binding energy Δ*E*_bind_ with various R-substituents for the strongly bound complexes of the RPH_2_···IF type. The graph includes comparison values for the alternative theory levels given in Tables S1–S3 of SI. From Figure 2, one can observe that significant quantitative differences in binding energies are calculated at the various theory levels, but the general trend with respect to the R-substituent variation is reasonably consistent in all cases. A slight exception is seen in the reduced cyano substituent effect in the “unmixed” B3LYP/LANL2DZ, MP2/LANLDZ theory levels, which may reflect the need for diffuse functions (present in the mixed-PP model but absent in uniform LANL2DZ) to better describe the strong electron-withdrawing effects of this substituent.

**Figure 3:**
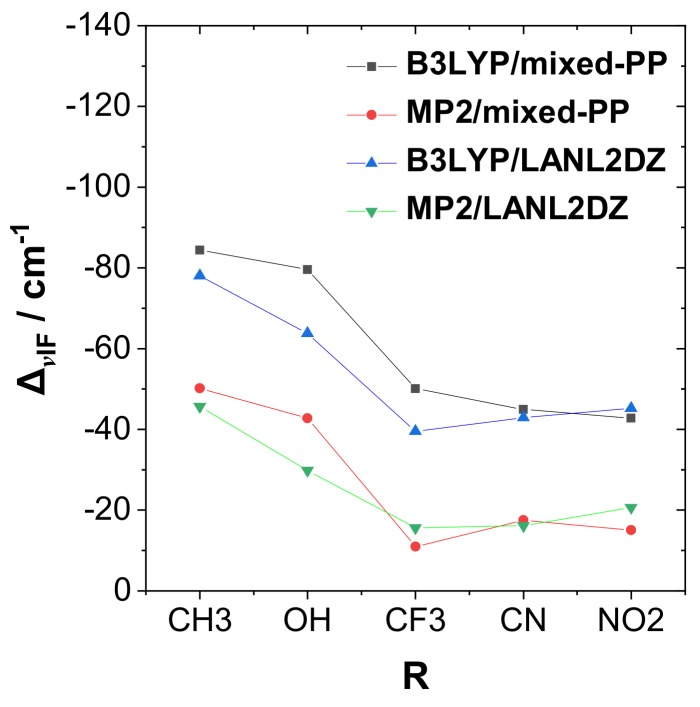
The trend of frequency shift, Δν_IF_, with the R-substituent in the RPH_2_···IF complexes for four theoretical levels (cf. Figure 2).

**Figure 4:**
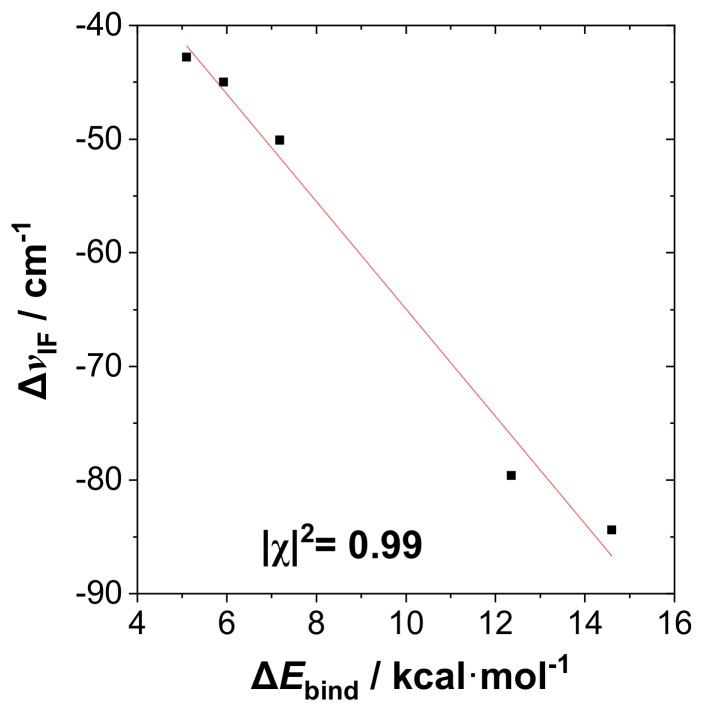
Correlation plot of frequency shift Δν_IF_ versus binding energy Δ*E*_bind_ for the RPH_2_···IF species (B3LYP/mixed-PP level), showing the excellent least-squares regression fit (dashed line) and Pearson correlation coefficient (|χ|^2^ = 0.99) for these experimentally measurable properties.

Figure 3 shows a corresponding plot of dihalogen frequency shift Δν_IF_ with respect to the R-substituent (including comparison results from alternative methods). As shown in the figure, Δν_IF_ is strongly red-shifted for the most strongly bound complexes, similar to the well-known spectroscopic signature of H-bonding. Figure 4 exhibits the correlation plot of Δν_IF_ (Figure 3) versus Δ*E*_bind_ (Figure 2) in the RPH_2_···IF species, showing the high correlation (Pearson |χ|^2^ = 0.99) between these experimentally observable quantities that confirms the similarity to H-bonding.

Returning to the structural data of Table 1 and Figure 1 for the strongly bound RPH_2_···IF complexes, one may note that the intermolecular P···I distances vary in the range 2.86–3.03Å. Table 2 displays empirical van der Waals (vdW) radii (Pauling values [59]) for the P, I, and F atoms of present interest, showing that the optimized *R*_P__···I_ distances in these complexes are all more than 1Å inside nominal van der Waals contact. This deep penetration into the exponentially repulsive sub-vdW region implies the presence of powerful intermolecular attractive forces (also of exponentially increasing strength) to achieve the net binding energies displayed in Table 1 and Figure 2. In addition, the circa 0.2Å variation in penetration distance with the R-substituent testifies to the impressive strength of substituent effects that further modulate Δ*E*_bind_ binding affinity and *R*_P__···I_ separation in the deep sub-vdW region.

The computed structural and binding characteristics noted above identify the propensities and patterns to be explained and provide helpful clues to the nature of the underlying attractive forces. Even the more weakly bound complexes of the P···FI type exhibit optimized P···F distances (2.1–2.6Å) that lie significantly (>0.5Å) inside vdW contact (circa 3.2Å, Table 2). Indeed, in both strong RPH_2_···IF and weak RPH_2_···FI complexes, the structural features hint at striking resemblances to familiar H-bonding phenomena, including deep sub-vdW penetration (with concomitant covalent bond *R*_IF_ elongation) and pronounced “linearization” tendency (albeit along different 3-center axes: P···IF in the RPH_2_···IF case versus RP···F in the RPH_2_···FI case). We take up NBO/NRT investigation of these apparent similarities and bonding relationships in the following sections.

## 3. NBO/NRT Descriptors

As suggested in (1a,b) above, an NBO/NRT analysis of B:···HA hydrogen-bonding phenomena reveals a host of characteristic correlations [22,23,60] that implicate resonance-type *n*_B_→σ*_AH_ “charge transfer” interactions as the fundamental electronic origin of H-bonding, consistent with the earliest results of NBO analysis [61,62,63]. This conclusion also supports a considerable variety of other experimental and theoretical evidence [64,65,66,67,68] for the charge–transfer nature of H-bonding. The question here is whether similar NBO/NRT diagnostics may provide analogous associations with *n*→σ* (lone pair to valence antibond) donor–acceptor interactions of the present species.

The analogies of the preceding section suggest appropriate NBO/NRT descriptors for the corresponding ···X— (“X-ogen”) species that can be similarly visualized in simple orbital overlap terms. For RPH_2_···IF complexes, the key feature of envisioned P···I—F “halogen bonding” is the donor–acceptor delocalization of electronic charge from an occupied donor NBO of P (lone pair *n*_P_) to the favorably polarized acceptor NBO (antibond σ*_IF_) of the P···I—F triad. For RPH_2_···FI complexes, where the *n*_P_-σ*_IF_ overlap is unfavorable, the analogous feature of envisioned F···P—R “pnicogen bonding” is the donation from a fluorine lone pair (*n*_F_) to the proximal antibond (σ*_PR_) of the phosphine monomer. Each such *n*→σ* delocalization can be alternatively quantified in terms of energetic (perturbative Δ*E*^(2)^_nσ*_ or variational deletion Δ*E*^($DEL)^_nσ*_), charge transfer (*Q*_nσ*_), or fractional NRT bond order (*b*_P···I_ or *b*_P···F_) descriptors.

### 3.1. Energy Descriptors

Table 3 summarizes estimates of the Δ*E*_nσ*_ stabilization energy associated with each type of delocalization in the strongly bound RPH_2_···IF species. The first two columns display 2^nd^-order perturbative Δ*E*^(2)^_nσ*_ estimates for *n*_P_→σ*_IF_ (“halogen bond”) and *n*_I_→σ*_PR_ (“pnicogen bond”) contributions. The final two columns of Table 3 display corresponding Δ*E*^($DEL)^_nσ*_ variational deletion estimates, obtained as the variational energy raising when the specific *n*→σ* NBO interaction is deleted from the total energy evaluation. The two estimates are seen to be in reasonable qualitative agreement for each interaction type. The Δ*E*^(2)^_n(P)→__σ*(IF)_ values range from 19 to 37 kcal/mol (in the same order as Δ*E*_bind_), whereas Δ*E*^(2)^_n(I)→__σ*(FI)_ is relatively negligible in each case, and a similar pattern is seen in the Δ*E*^($DEL)^_nσ*_ values. We therefore expect that the stabilization energy of the *n*_P_→σ*_IF_ interaction is the dominant attractive contribution to the structure and binding of RPH_2_···IF complexes, similar to the dominance of the *n*_B_→σ*_AH_ interaction in H-bonding.

For the weakly bound RPH_2_···FI species, the situation is more complex. Due to the unfavorable polarization of σ*_FI_ away from the *n*_P_ lone pair, the single *n*_P_→σ*_IF_ interaction that dominates bonding in RPH_2_···IF species becomes only one of several such competing contributions to net binding. Specifically, for the opposed F···P—R (pnicogen bonding) motif, three possible *n*_F_ lone pairs (one of sigma type and two of pi type) and three σ*_PY_ acceptors (one σ*_PR_ and two σ*_PH_) are within proximal interaction range as potential contributors to a complex resonance mixture. A particular interaction such as *n*_F_^(σ)^→σ*_PR_ therefore represents only one of the nine related *n*→σ* interactions that may exert leverage on angular structure and binding energy. In such a case, only a more nuanced resonance-type description (see the discussion of NRT bond orders below) can describe the complete bonding picture that provides useful correlations with experimentally measurable properties. We therefore defer further discussion of RPH_2_···FI complexes to a later subsection.

### 3.2. Graphical Orbital Overlap Imagery

A powerful aspect of NBO energetic descriptors is their intimate relationship to the intuitive visual imagery of the orbital overlap, the NBO counterpart of the well-known “Mulliken approximation” [69,70,71,72] that underlies semi-empirical MO theory. For such visualization purposes, “pre-orthogonal” NBOs (PNBOs) [49] are employed that maintain free-space symmetries (cartoon-like “textbook” shapes) of non-interacting NBOs prior to the actual distortions of the molecular environment. Table 4 displays such PNBO visualizations for the various donor–acceptor *n*→σ* interactions of halogen (*n*_P_→σ*_IF_; first column) or pnicogen (*n*_I_^(π)^→σ*_PR_, *n*_I_^(σ)^→σ*_PR_; final two columns) types, with corresponding numerical Δ*E*^(2)^_nσ*_ values (Table 3) inset in the panels.

For example, in the three panels for CH_3_PH_2_···IF (first row of Table 4) one can see that the “end-on” (sigma-type) overlap of σ*_IF_ with the nitrogen lone pair *n*_P_ (left panel) is much stronger than the “edge-on” (pi-type) overlap of σ*_PR_ with the in-plane iodine *n*_I_^(π)^ (middle panel) or *n*_I_^(σ)^ (right panel) lone pairs, in accordance with the Δ*E*^(2)^_nσ*_ values shown in each panel. Even a casual glance at the overall patterns of the orbital overlap in Table 4 can therefore make clear why the slight variations of orbital shape with R-substitution do not materially alter the strong propensity for halogen-type (*n*_P_→σ*_IF_) rather than pnicogen-type (*n*_I_^(π)^→σ*_PR_, *n*_I_^(σ)^→σ*_PR_) bonding throughout the RPH_2_···IF series, as numerically displayed in Table 3.

Though the donor–acceptor Δ*E*_nσ*_ stabilization energies of Table 3 display a qualitative ordering pattern similar to that of the Δ*E*_bind_ binding energies of Table 1, it is evident that these estimates significantly “overshoot” the final net binding energy. This is to be expected, because each stabilizing donor–acceptor interaction is opposed by steric repulsions of the corresponding Δ*E*_nσ_ donor–donor interaction. Table 5 displays orbital overlap diagrams and inset values of the pairwise estimates Δ*E*^(pw)^_n__-__σ_ for repulsive “steric exchange energy” [54] between occupied NBOs of the RPH_2_···IF series. As shown in the panels, the two leading intermolecular repulsions refer to the *n*_P_-*n*_I_ steric clash between lone pairs of proximal P and I atoms (left) and *n*_P_-σ_IF_ clash between a lone pair and bond of the halogen bond P···I—F triad. However, weaker *n*_I_-σ_PR_, *n*_I_-σ_PH_ clashes of the alternative pnicogen bond triads (I···P—R, I···P—H) also contribute to sub-vdW steric opposition, thereby further offsetting the apparently “excessive” NBO estimate of donor–acceptor attraction needed to achieve the final equilibrium geometry and net binding energy.

PNBO visualizations for occupied *n*_P_, *n*_I_, and σ_IF_ NBOs (not shown) similarly serve to rationalize the trends in repulsive donor–donor interactions (Table 5). Furthermore, it becomes evident from graphical comparisons that all such orbital visualizations for the presently considered species are highly analogous to those for the corresponding interactions of H-bonding [22], which is in accordance with the patterns and relationships inferred from numerical descriptors.

### 3.3. Structural, Energetic, and Spectroscopic Effects of Donor–Acceptor Deletion

Still another widget from the NBO toolbox can be used to exhibit the profound effects of *n*_P_→σ*_IF_ delocalization on supramolecular bonding properties. Similar to the variational $DEL-deletion Δ*E*^($DEL)^_n(P)→σ*(IF)_ evaluations of *n*→σ* donor–acceptor strength in the equilibrium species (Table 3), one can variationally reoptimize the structure for the associated *E*^($DEL)^_n(P)→__σ*(IF)_ potential energy surface in which *n*→σ* delocalization is absent, as though nature (in accordance with common textbook presentations) failed to include such interactions.

Table 6 displays results for the variational reoptimization of a simple 2D model of RPH_2_···IF dissociation, with the *n*_P_→σ*_IF_ interaction deleted. Starting from the equilibrium values of the actual RPH_2_···IF species, the distance variables *R*_P···I_, *R*_IF_ (for fixed idealized angular geometry) were optimized to find the new binding energy (Δ*E*_bind_) and shifts (Δ*R*_P···I_, Δ*R*_IF_, ΔΔ*E*_bind_**)** that describe the hypothetical “resonance-free” RPH_2_···IF that lacks *n*_P_→σ*_IF_ donor–acceptor attraction. From the resulting values, one sees that characteristic structural and energetic signatures of RPH_2_···IF bonding are lost when the *n*_P_→σ*_IF_ interaction is “turned off;” the RPH_2_ and IF monomers retreat (by circa 1.3Å) to beyond-vdW separation, the characteristic *R*_IF_ elongation (associated with vibrational red-shifting) from isolated monomer geometry essentially disappears, and the net binding energy drops precipitously (by >80%) to a remnant value that might plausibly be associated with residual “dipole–dipole” forces. Note that the monomer dipole moments or other aspects of electron density distribution are scarcely altered in the $DEL-reoptimized geometry, and the “lost” energy of *n*_P_→σ*_IF_ stabilization is negligibly small (1.59 kcal/mol) at the reoptimized separation distance. From the results of such $DEL-deletion calculations, one recognizes *n*_P_→σ*_IF_ stabilization to be the unique “smoking gun” that is both necessary and sufficient to bring the supramolecular complex to the actual short-range geometry and other signatures of halogen bonding, which is in direct correspondence to the analogous *n*_B_→σ*_HA_ role in H-bonding.

**Figure 5:**
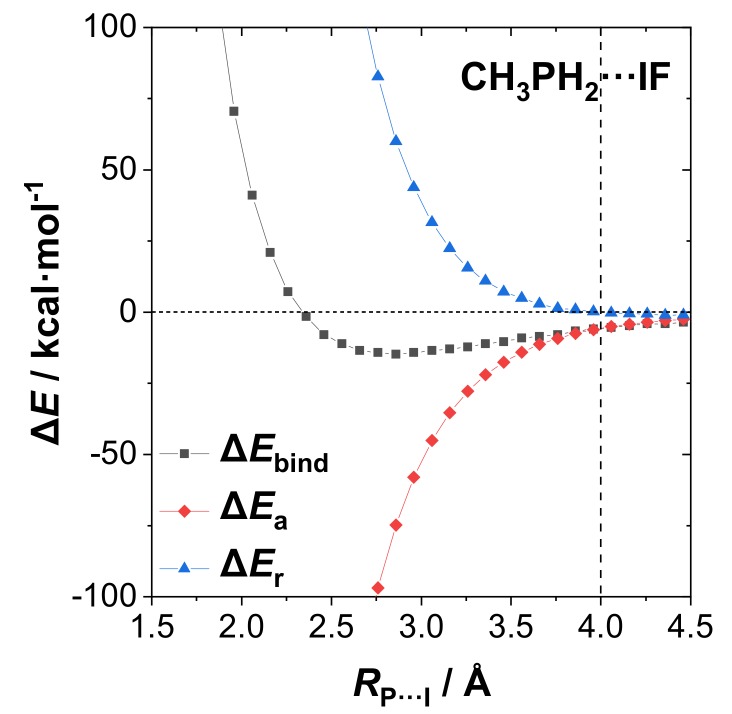
Potential curves for CH_3_PH_2_···IF dissociation (B3LYP/mixed-PP level), showing (i) repulsive “resonance-free” potential Δ*E*_r_ (blue, triangles) with all resonance-type intermolecular delocalizations deleted, (ii) the stabilizing potential for attractive resonance-type interactions Δ*E*_a_ (red, diamonds) that were deleted in the resonance-free model, and (iii) the combined “full” potential Δ*E*_bind_ (sum of (i), (ii)) (black, squares).

Still another use of $DEL-deletion techniques is to prepare relaxed-scan potential curves that illustrate how intermolecular *n*→σ* interactions lead to net binding against the essentially repulsive potential curve for hypothetical monomers that interact without benefit of resonance-type stabilization. Figure 5 illustrates such potential curves for CH_3_PH_2_···IF dissociation, showing the potential curve for the fully interacting monomers (squares, black line, “Δ*E*_bind_”; cf. Table 1) versus that for “resonance-free” monomers that lack intermolecular *n*→σ* interactions (triangles, blue line, repulsive “Δ*E*_r_”). The “missing” resonance-type (purely attractive) interactions are shown as the difference curve (diamonds, red line, attractive “Δ*E*_a_”). The blue curve exhibits a feeble attractive well beyond vdW contact at *R*_P···I_ ≈ 4.0Å (presumably due to “dipole–dipole” attraction) but rises steeply as a repulsive steric wall in the sub-vdW region. Already in the region of the initial sub-vdW penetration, the red curve (*n*→σ* resonance attraction) has achieved sufficient stabilization to oppose thermal fluctuations (*kT* ≈ 0.6 kcal/mol at 300K) and yield significant sub-vdW binding in the black (full potential) curve down to *R*_P···I_ ≈ 2.9 equilibrium separation. Inside this equilibrium distance, the intermolecular *n*-σ steric repulsion overcomes *n*-σ* attraction to give the steeply repulsive inner wall of the full potential, resonance-shifted inside circa 2.5Å. Where such *n*_P_-σ_IF_ versus *n*_P_-σ*_IF_ “cross-over” occurs will evidently depend on the polarization of the dihalogen bond and is therefore expected to shift to a progressively larger *R*_P···I_ separation (and weakened Δ*E*_bind_) for other members of the dihalogen series, thereby appearing as a secondary “electrostatic” modulating effect on the overall halogen bonding phenomenon.

### 3.4. Charge and Polarity Descriptors

Table 7 displays various NBO charge/polarization descriptors for the RPH_2_···IF species (net intermolecular charge transfer *Q*_CT_, atomic charges *q*_P_, *q*_I_, and *q*_F_ of the P···I—F triad, IF bond ionicity *i*_IF_ [73]), compared with those for isolated IF monomer. As shown for the dihalogen monomer in the final column of the table, I–F bond ionicity (−1 ≤ *i*_IF_ ≤ +1, related to dipole moment direction from I to F) tends to increase steadily with strength of RPH_2_···IF binding or net *Q*_CT_. This trend reflects the expected dominance of the *n*_P_→σ*_IF_ interaction, which is evidently enhanced (cf. overlap diagrams of Table 5) if σ_IF_ bond ionicity increases toward F, thereby polarizing the σ*_IF_ antibond toward I (and adjacent *n*_P_). The combined effect of repolarization (*i*_IF_) and net charge transfer (*Q*_CT_) is to give a somewhat irregular pattern to individual atomic charges *q*_I_, *q*_F_, but all such IF charge descriptors are seen to properly “add up,” as they must according to the strict logic of a natural population analysis.

In the RPH_2_ monomer, the phosphorus charge *q*_P_ reflects the still more complex effects of sigma-type induction versus intra- and intermolecular lone pair delocalizations. One can see in the table the evident effect of bonding the phosphorus to more electronegative oxygen (*q*_P_ ≈ 0.73) or nitrogen (*q*_P_ ≈ 0.45) rather than carbon (*q*_P_ ≈ 0.3–0.4). However, also important in the present context is the intra- versus intermolecular competition for charge donation from the “busy” phosphorus lone pair *n*_P_ to various pi-type acceptor orbitals of the R-substituent (e.g., π_CN_, π_NO_) versus the intermolecular *n*_P_→σ*_IF_ interaction of principal interest.

### 3.5. NRT Bond Order Descriptors

Perhaps the most useful and general descriptors of supramolecular bonding are NRT bond orders {*b*_AB_}, which balance the nuances of multiple resonance structure contributions to give a single composite measure of A···B “connectivity.” As described elsewhere [40,41,42,43], NRT bond orders differ from those of the Pauling type, particularly in their close association with the first-order reduced density matrix [74] (rather than assumed valence bond (VB) wavefunction) of the system. A vastly improved convex solver [75] for the NRT variational objective function is a leading feature of the *NBO 7.0* program [57].

The first three columns of Table 8 show calculated NRT bond orders for the key P/I/F atoms in both the RPH_2_···IF and RPH_2_···FI series. The values vary rather continuously over the range 0 ≤ *b*_AB_ ≤ 1, with no apparent “dividing line” between what should be described as the “covalent bond” (stick) versus the “noncovalent interaction” (dots). Most surprising in these species are the non-vanishing bond orders for the “long-bond” [76,77,78] interactions of P^F (in P···I—F triads) or P^I type (in P···F—I triads). Though negligibly small in the most strongly bound RPH_2_···IF species, certain P^I long bonds achieve bond orders that rival or exceed those of familiar *b*_P···F_ or *b*_IF_ linkages (e.g., in OHPH_2_···FI).

The final two columns of Table 8 show two-term or three-term bond order sums that test the validity of resonance-type “bond order conservation” [48] rules (cf. (1a,b) for H-bonding). The two-term sum (column 4) exhibits significant deviations from the expected unit value in a simple two-resonance model for a mono-valent central atom of the 3c/4e triad. These deviations indicate the need for a three-term sum (three-resonance model) that includes long-bond contributions (column 5). Remaining deviations from unity in the three-term sums of column 5 indicate additional conjugative or hyperconjugative interactions with the R-substituent that require still higher-order resonance couplings. All these results suggest the close relationship of NRT bond orders to the familiar resonance-theoretic concepts of the organic chemist, as well as their usefulness in expected empirical correlations with experimentally measurable structural, energetic, and spectroscopic properties, as discussed in the following section.

## 4. Mutual Correlations of NBO/NRT Descriptors with Measurable Properties

The principal NBO descriptors of donor–acceptor interactions (such as Δ*E*^(2)^_nσ*_, Δ*E*^($DEL)^_nσ*_, *Q*_nσ*_,...) intrinsically focus on orbitals of a specific *n*→σ* “delocalization” from a specific Lewis structural bonding pattern. Such orbital-specific descriptors are valuable when a single “parent” Lewis structure and “child” *n*→σ* delocalization clearly dominate quantum mechanical descriptions of the structural, energetic, and spectroscopic properties of the chosen system. However, for many chemical systems of interest (including the RPH_2_···FI species considered here), multiple *n*→σ* interactions come into play, corresponding to contributions of alternative resonance–structural bonding patterns that may no longer be “child-like” compared to a reference parent pattern. Such cases demand the more nuanced NRT descriptors such as interatomic bond orders {*b*_AB_} that balance the many possible orbital interactions contributing to the overall resonance hybrid.

As could be anticipated from the empirical origins of bond order descriptors in the pre-quantum mechanical conceptions of “mesomerism” theory [43], the “averaged” (fractional) character of NRT bond orders {*b*_AB_} and their association with atoms (rather than orbitals) makes them better adapted to describe empirical correlations with measurable properties. Well-known examples include bond order–bond length (*b*_AB_-*R*_AB_) [79], bond order–bond energy [80], and bond order–bond frequency (Badger’s rule) [81] relationships. Here we wish to briefly examine the mutual correlations of NBO/NRT descriptors in the single strong *n*→σ* limit (where orbital-type descriptors may suffice) as well as the more general correlations of NRT bond orders with experimentally measurable properties, spanning the full set of RPH_2_···IF/FI species considered in this work.

**Figure 6:**
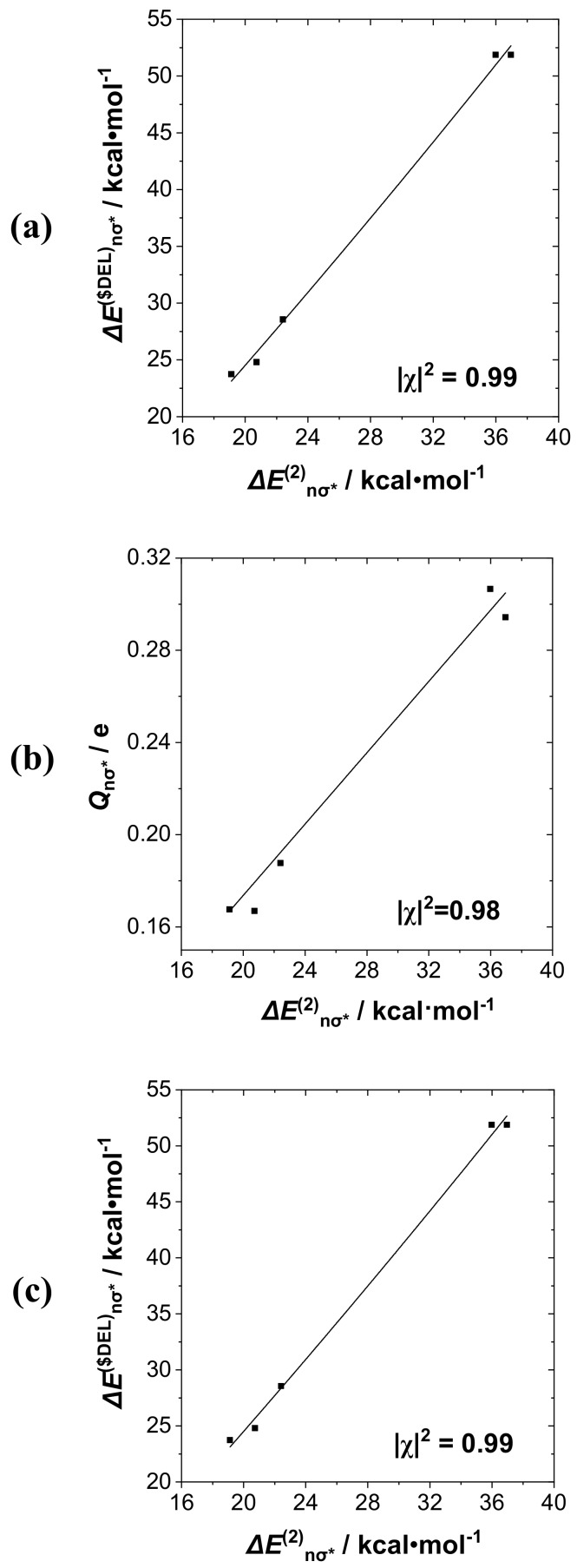
Correlation diagrams for (**a**) Δ*E*^(2)^_nσ*_-Δ*E*^($DEL)^_nσ*_, (**b**) Δ*E*^(2)^_nσ*_-*Q*_nσ*_, and (**c**) Δ*E*^(2)^_nσ*_-*b*_P···I_ descriptors of RPH_2_···IF species, showing least-squares regression line and Pearson correlation coefficient |χ|^2^ for each pairing.

Starting with the RPH_2_···IF series, in which the *n*_P_→σ*_IF_ interaction plays a clearly dominant role, we first consider the mutual correlations of Δ*E*^(2)^_nσ*_, Δ*E*^($DEL)^_nσ*_, *Q*_nσ*_, *b*_P···I_ descriptors for this interaction. Taking perturbative Δ*E*^(2)^_nσ*_ as the base descriptor for the group, we display (**a**) Δ*E*^(2)^_nσ*_-Δ*E*^($DEL)^_nσ*_, (**b**) Δ*E*^(2)^_nσ*_-*Q*_nσ*_, and (**c**) Δ*E*^(2)^_nσ*_-*b*_P···I_ correlation diagrams in successive panels (**a**)–(**c**) of Figure 6. The displayed least-squares regression lines and Pearson |χ|^2^ correlation coefficients demonstrate the high quality of these mutual correlations: In the range 0.94–0.99 for NBO-specific descriptors, but slightly lower (0.94) for correlation with the NRT bond order *b*_P···I_ (presumably due to the proper inclusion of secondary donor–acceptor interactions with R-substituents that are present only in the latter). The results show that descriptors of this group could be chosen rather interchangeably for correlations with measurable experimental properties when a single donor–acceptor interaction such as *n*_P_→σ*_IF_ is clearly dominant.

**Figure 7:**
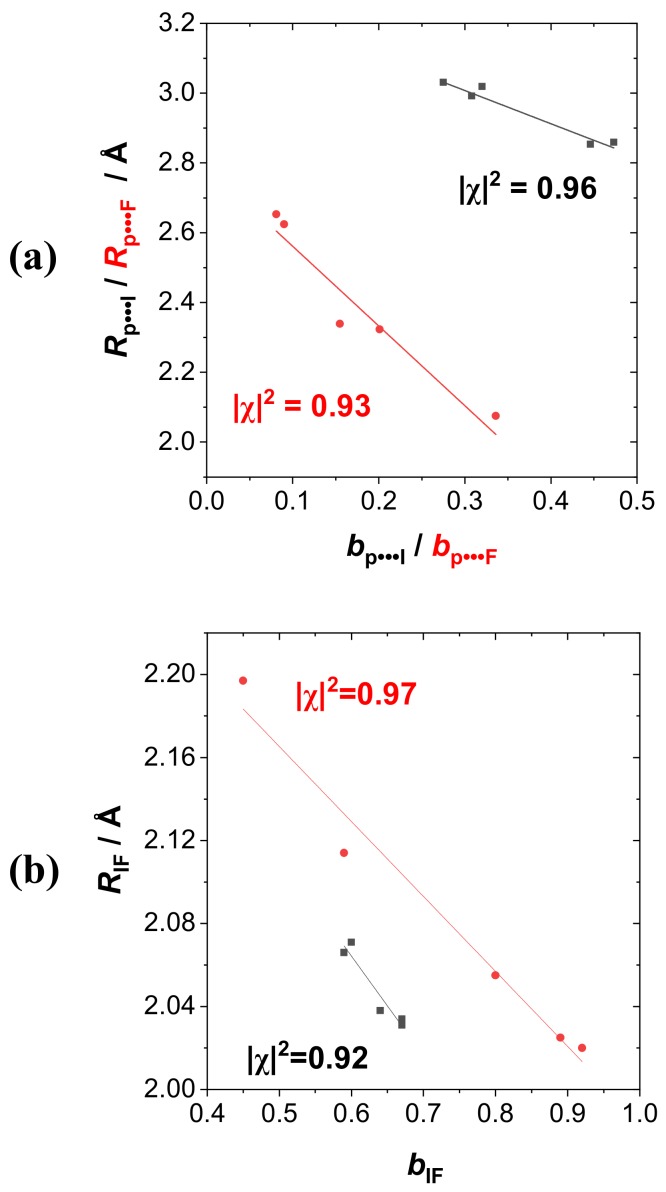

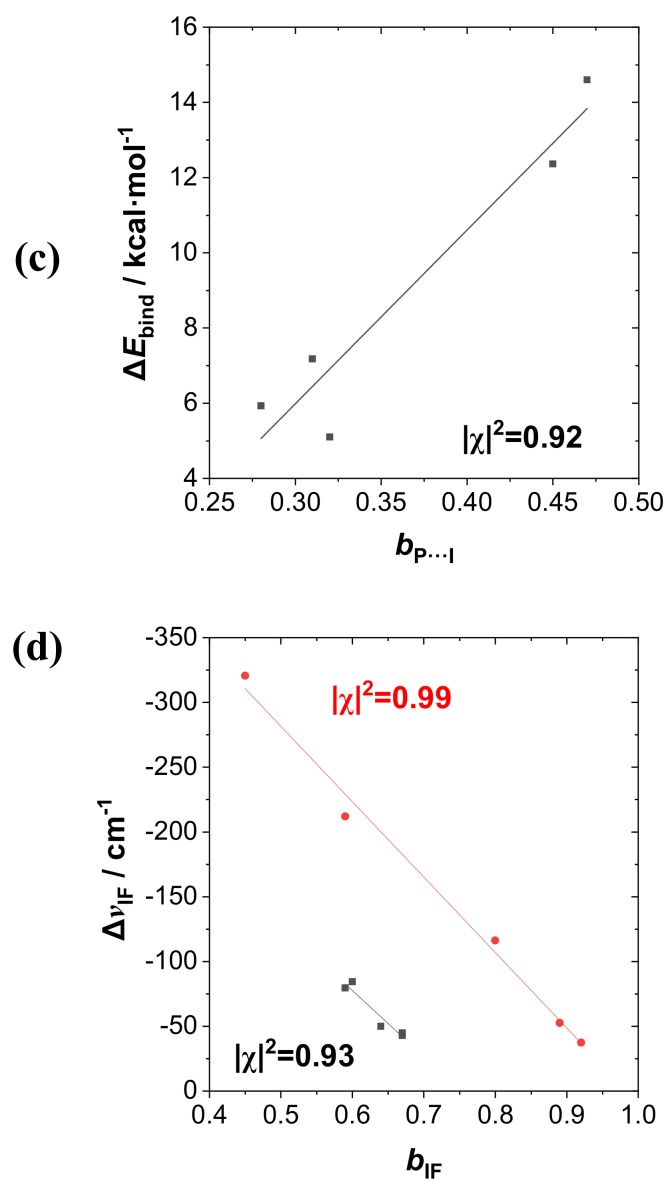
Bond order–bond property correlation diagrams for (**a**) *b*_P···X_-*R*_P···X_, (**b**) *b*_IF_-*R*_IF_, (**c**) *b*_P···X_-Δ*E*_bind_, and (**d**) *b*_IF_-Δν_IF_ descriptors of RPH_2_···IF (black) and RPH_2_···FI (red) species, showing least-squares regression line and Pearson correlation coefficient |χ|^2^ for each pairing.

For more general correlations with measurable experimental properties such as (a) intermolecular *R*_P···I_ or *R*_P···F_, (b) intramolecular *R*_IF_, (c) binding energy Δ*E*_bind_, or (d) infrared stretching frequency ν_IF_, we employed the NRT bond order *b*_AB_ (of specified atoms A, B) as base descriptor in the correlation diagrams of Figure 7a–d, extending the correlations to both RPH_2_···IF (black; approximate two-resonance) and RPH_2_···FI (red: Multi-resonance) series. As examples of bond order­bond length correlations, Figure 7a displays the intermolecular *b*_P···X_-*R*_P···X_ correlations (X = I, F) for each series, with reasonably high correlations (|χ|^2^ = 0.92) in each case. Figure 7b similarly displays the intramolecular *b*_IF_-*R*_IF_ correlation for the dihalogen monomer, with still higher correlation coefficients (|χ|^2^ = 0.94 for RPH_2_···IF, |χ|^2^ = 0.98 for RPH_2_···FI species). As examples of bond order–bond energy correlation, Figure 7c displays the *b*_P···X_-Δ*E*_bind_ correlations (X = I, F), with similar correlation coefficients (|χ|^2^ = 0.94) in each series. Finally, as bond order–bond frequency examples, Figure 7d displays the *b*_IF_-Δν_IF_ correlations, again with high correlation coefficients (|χ|^2^ = 0.95 for RPH_2_···IF, |χ|^2^ = 0.98 for RPH_2_···FI species). All these correlations suggest the high predictive utility of NRT bond orders for both the tuning effects of R-substituents and the polarity variations of the various dihalogen monomers that govern the broad range of binding energies in these supramolecular species.

## 5. Summary and Conclusions

We have computationally investigated the nature of supramolecular bonding in a series of R-substituted phosphine···dihalogen complexes (RPH_2_···IF/FI, R = CH_3_, OH, CF_3_, CN, NO_2_), focusing on the electronic origin of what might be identified as a “halogen bond,” “pnicogen bond,” or some combination of both. For this purpose, we employed a broad variety of natural bond orbital (NBO) and natural resonance theory (NRT) descriptors, searching for relationships to hydrogen bonding or other known forms of intermolecular attraction (often loosely termed “noncovalent”).

Our results show that resonance-type “*n*→σ*” delocalization (leading to fractional intermolecular bond orders) is the dominant feature of halogen-type (e.g., *n*_P_→σ*_IF_) or pnicogen-type (e.g., *n*_F_→σ*_PR_) bonding motifs, just as for the dominant *n*_B_→σ*_HA_ interaction of B:···H—A hydrogen bonding. We obtained evidence both for resonance-type “bond conservation” rules and the complete set of mutually consistent correlations of NBO/NRT descriptors with experimentally measurable properties of RPH_2_···IF/FI complexes that closely parallel those previously demonstrated for H-bonded complexes. We also demonstrate that the removal of *n*→σ* interactions obliterates the signature features of halogen/pnicogen bonding, whereas such features persist (albeit in muted form) even if envisioned “dipole–dipole” contributions are reversed (by reversing dihalogen monomer direction). Thus, our results establish that resonance-type *n*→σ* stabilization is both necessary and sufficient for the characteristic structural, energetic, and spectroscopic features of halogen or pnicogen bonding, as was previously demonstrated for H-bonding [22,23]. The present results are fully consistent with previous studies of orbital-interaction effects in halogen or pnicogen bonding [82,83]. Connections can also be seen to more nuanced interpretation of the “σ-hole” in terms of chemical “lone pair” interactions with the “extension of one of the covalent bonds,” “dative sharing (coordinate covalence),” and other quantal phenomena [84].

Based on these results, we anticipate that similar unique associations with resonance-type *n*→σ* “fractional bonding” will be found for chalcogen bonds, tetrel bonds, and all other such supramolecular bonding phenomena. Our conclusions thereby extend “resonance covalency” concepts to the entire supramolecular domain of sub-integer bond orders, challenging the common “electrostatic” assumptions that underlie current empirical force field modeling and textbook expositions of supramolecular chemistry. 

## Figures and Tables

**Table 1:** Optimized dihalogen RPH_2_···IF and RPH_2_···FI complexes (B3LYP/mixed-PP level), showing key structural and energetic descriptors: Bond lengths (*R*_P···X_, *R*_IF_, *R*_RP_; Å) and changes from monomer values (Δ*R*_IF_, Δ*R*_RP_; Å), orientation angles (Θ_RPX_, Θ_PXY_; degrees), dihalogen infrared frequency shift (Δν_IF_; cm^−1^), and binding energy (Δ*E*_bind_; kcal mol^−1^).

RPH_2_···IF	*R*_P···I_	*R*_IF_	Δ*R*_IF_	*R*_RP_	Δ*R*_RP_	Θ_RPI_	Θ_PIF_	Δν_IF_	Δ*E*_bind_
CH_3_PH_2_···IF	2.859	2.071	0.064	1.845	−0.027	115.4	178.2	−84.4	14.60
OHPH_2_···IF	2.854	2.066	0.059	1.652	−0.036	117.1	179.2	−79.6	12.36
CF_3_PH_2_···IF	2.992	2.038	0.031	1.894	−0.002	122.1	177.9	−50.1	7.18
CNPH_2_···IF	3.031	2.034	0.027	1.783	−0.014	121.2	177.6	−45.0	5.93
NO_2_PH_2_···IF	3.019	2.031	0.024	1.880	0.000	123.1	175.3	−42.8	5.10
**RPH_2_···FI**	***R*_P···F_**	***R*_IF_**	**Δ*R*_IF_**	***R*_RP_**	**Δ*R*_RP_**	**Θ_RPF_**	**Θ_PFI_**	**Δν_IF_**	**Δ*E*_bind_**
CH_3_PH_2_···FI	2.323	2.114	0.107	1.873	0.001	166.1	164.4	−211.9	3.25
OHPH_2_···FI	2.075	2.197	0.190	1.684	−0.004	164.8	157.4	−320.6	5.85
CF_3_PH_2_···FI	2.624	2.025	0.018	1.900	0.004	172.7	165.1	−52.6	4.43
CNPH_2_···FI	2.653	2.020	0.013	1.805	0.008	173.1	164.6	−37.3	4.85
NO_2_PH_2_···FI	2.339	2.055	0.048	1.902	0.022	177.1	160.0	−116.2	6.79

**Table 2:** Van der Waals radii of P, I and F atoms (Pauling, Ref. [59]).

Atom	P	I	F	P + I	P + F	I + F
**VDW radii/Å**	1.9	2.15	1.35	4.0	3.2	3.5

**Table 3:** Δ*E*_nσ*_ stabilization energy estimates(kcal/mol) for halogen-type [*n*(P)→σ*(IF)] and leading pnicogen-type [*n*(I)→σ*(PR)] interactions of RPH_2_···IF complexes, showing perturbative Δ*E*^(2)^_nσ*_ values in the first two columns and variational deletion Δ*E*^($DEL)^_nσ*_ values in the final two columns (The connected R-atom of each σ*_PR_ acceptor NBO is shown underlined in the species listing.).

Species	Δ*E*^(2)^_n(__P)→σ*(__IF)_	Δ*E*^(2)^_n(__I)→σ*(__PR)_	Δ*E*^($DEL)^_n(__P)→σ*(__IF)_	Δ*E*^($DEL)^_n(__I)→σ*(__PR)_
CH_3_PH_2_···IF	36.96	1.32	51.87	1.47
OHPH_2_···IF	35.99	2.12	51.88	2.60
CF_3_PH_2_···IF	22.42	1.62	28.56	1.69
CNPH_2_···IF	20.73	1.40	24.80	1.73
NO_2_PH_2_···IF	19.12	2.69	23.73	2.64

**Table 4:** “Pre-orthogonal” natural bond orbital (PNBO) orbital overlap diagrams (with perturbative Δ*E*^(2)^_nσ*_ stabilization energy (kcal/mol) as inset) for RPH_2_···IF complexes (cf. Table 3), showing the usefulness of Mulliken-type orbital overlap visualizations in “guesstimating” donor–acceptor interaction strengths.

Species	*n*_P_→σ*_IF_	*n*_I_^(π)^→σ*_PR_	*n*_I_^(σ)^→σ*_PR_
CH_3_PH_2_···IF	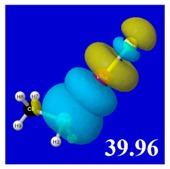	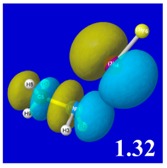	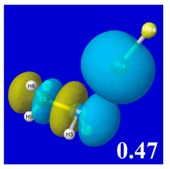
OHPH_2_···IF	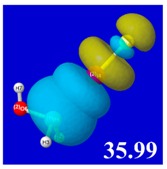	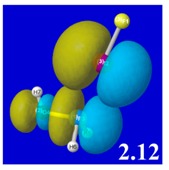	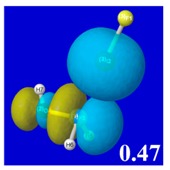
CF_3_PH_2_···IF	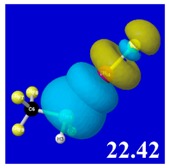	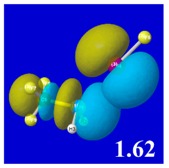	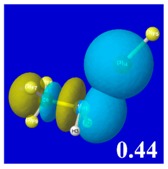
CNPH_2_···IF	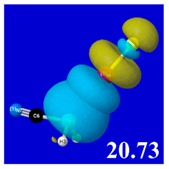	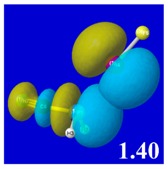	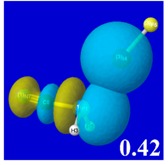
NO_2_PH_2_···IF	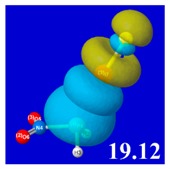	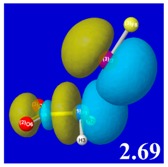	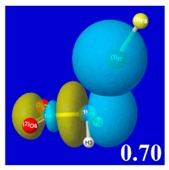

**Table 5:** Δ*E*^(pw)^_donor–donor_ repulsion energy estimates (kcal/mol) for leading *n*_P_-*n*_I_ and *n*_P_-σ_IF_ steric clashes of RPH_2_···IF complexes, obtained from steric-keyword analysis.

Species	Δ*E*^(pw)^_n(__P)-n(__I)_	Δ*E*^(pw)^_n(__P)-σ(__IF)_
CH_3_PH_2_···IF	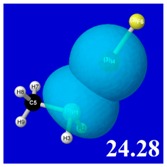	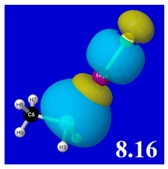
OHPH_2_···IF	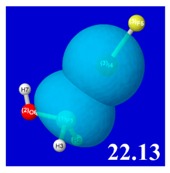	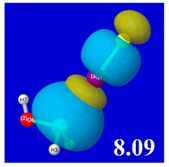
CF_3_PH_2_···IF	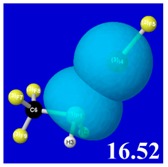	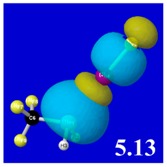
CNPH_2_···IF	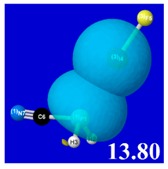	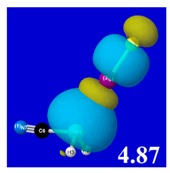
NO_2_PH_2_···IF	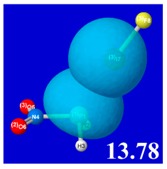	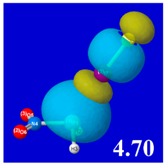

**Table 6:** Relaxed geometrical parameters (*R*_P···I_, *R*_IF_; Å), binding energy (Δ*E*_bind_; kcal/mol), and dihalogen stretching frequency (*ν*_IF_; cm^−1^) (with associated shifts from true equilibrium structure in parentheses) for $DEL-type variational reoptimizations, with the *n*_P_→σ*_IF_ charge–transfer interaction deleted.

Species	*R*_P···I_ (Δ*R*_P···I_)	*R*_IF_ (Δ*R*_IF_)	Δ*E*_bind_ (ΔΔ*E*_bind_)
CH_3_PH_2_···IF	4.20 (1.34)	2.007 (−0.064)	2.32 (−12.28)
OHPH_2_···IF	4.29 (1.43)	2.006 (−0.060)	1.31 (−11.05)
CF_3_PH_2_···IF	4.34 (1.35)	2.003 (−0.035)	0.96 (−6.22)
CNPH_2_···IF	4.37 (1.34)	2.003 (−0.031)	0.63 (−5.30)
NO_2_PH_2_···IF	4.40 (1.39)	2.003 (−0.028)	0.38 (−4.72)

**Table 7:** Total RPH_2_ → IF charge transfer (*Q*_CT_; *e*), atomic charges (*q*_P_, *q*_I_, *q*_F_), and σ_IF_ bond ionicity (*i*_IF_) in RPH_2_···IF complexes, compared with isolated IF monomer.

Species	*Q*_CT_	*q*_P_	*q*_I_	*q*_F_	*i*_IF_
CH_3_PH_2_···IF	0.2405	0.4084	0.3227	−0.5632	0.496
OHPH_2_···IF	0.2432	0.7345	0.3144	−0.5576	0.486
CF_3_PH_2_···IF	0.1413	0.2974	0.3857	−0.5270	0.485
CNPH_2_···IF	0.1255	0.3493	0.3924	−0.5179	0.480
NO_2_PH_2_···IF	0.1137	0.4496	0.4013	−0.5150	0.478
IF	-	-	0.4691	−0.4691	0.469

**Table 8:** Natural resonance theory (NRT) bond orders and bond order sums for the *b*_P···I_ halogen bond, *b*_IF_ covalent bond, and *b*_P^F_ long bond of RPH_2_···IF complexes (P···IF triad; upper rows) or the *b*_P···F_ pnicogen bond, *b*_IF_ covalent bond, and *b*_P^I_ long bond of RPH_2_···FI complexes (F···PR triad; lower rows). Note the near-constant “bond conservation” of the summed bond orders within each 3c/4e triad (final column).

Species	*b*_P···I_	*b*_IF_	*b*_P^F_	*b*_P···I_ + *b*_IF_	*b*_P···I_ + *b*_IF_ + *b*_P^F_
CH_3_PH_2_···IF	0.47	0.60	0.00	1.07	1.07
OHPH_2_···IF	0.45	0.59	0.00	1.04	1.04
CF_3_PH_2_···IF	0.31	0.64	0.11	0.95	1.05
CNPH_2_···IF	0.28	0.67	0.10	0.94	1.04
NO_2_PH_2_···IF	0.32	0.67	0.10	0.99	1.09
**Species**	***b*_P···F_**	***b*_IF_**	***b*_P^I_**	***b*_P···F_ + *b*_IF_**	***b*_P···F_ + *b*_IF_ + *b*_P^I_**
CH_3_PH_2_···FI	0.20	0.59	0.27	0.80	1.06
OHPH_2_···FI	0.34	0.45	0.36	0.79	1.14
CF_3_PH_2_···FI	0.09	0.89	0.07	0.98	1.05
CNPH_2_···FI	0.08	0.92	0.05	1.00	1.05
NO_2_PH_2_···FI	0.16	0.80	0.12	0.96	1.07

## Supplementary Materials

The following are available online. (1) Optimized geometrical parameters of title species for three alternative basis levels and the dispersion-corrected B3LYP-D3/mix-PP alternative method; (2) graphical displays of RPH_2_···FI complexes compared for all five alternative theory levels; (3) sample input files for NBO, STERIC, and DELETE-type calculations; and (4) optimized coordinates of all dihalogen complexes at the B3LYP/mixed-PP level.
