# Supplementary material for: What Is the Nature of Supramolecular Bonding? Comprehensive NBO/NRT Picture of Halogen and Pnicogen Bonding in RPH2···IF/FI Complexes (R = CH3, OH, CF3, CN, NO2)
Source: Molecules. 2019 May 31;24(11):2090. doi: 10.3390/molecules24112090 (PMC6600247; doi:10.3390/molecules24112090)
Supplement: Supplementary file 1 [file molecules-24-02090-s001.pdf]

Supporting Information

What is the Nature of Supramolecular Bonding?
Comprehensive NBO/NRT Picture of Halogen and Pnicogen Bonding in $RPH_2\cdots IF/FI$ Complexes ($R = CH_3, OH, CF_3, CN, NO_2$)

Yinchun Jiao[a] and Frank Weinhold[b]

[a]*Key Laboratory of Theoretical Organic Chemistry and Functional Molecules, Ministry of Education, School of Chemistry and Chemical Engineering, Hunan University of Science and Technology, Xiangtan, 411201, China;* [b]*Theoretical Chemistry Institute and Department of Chemistry, University of Wisconsin-Madison, Madison WI 53706*

Contents

1. Optimized parameters with other four methods……………………………………S2

2. Optimized graphics of $XH_2P\cdots FI$ complexes with five methods……….............S6

3. Examples of NBO, STERIC and DELETE input files of $CH_3PH_2\cdots IF$ complex....S10

4. Calculated coordinates of all complexes……………………………...……......S15

$RPH_2\cdots IF$	$R_{P\cdots I}$	R_{IF}	ΔR_{IF}	R_{RP}	ΔR_{RP}	Θ_{RPI}	Θ_{PIF}	$\Delta \nu_{IF}$	ΔE_{bind}
$CH_3PH_2\cdots IF$	2.948	2.053	0.039	1.834	-0.022	112.9	177.1	492.63	11.14
$OHPH_2\cdots IF$	2.993	2.045	0.031	1.653	-0.027	117.5	179.2	500.01	8.14
$CF_3PH_2\cdots IF$	3.209	2.023	0.009	1.874	-0.003	118.2	179.7	525.33	5.38
$CNPH_2\cdots IF$	3.246	2.021	0.007	1.782	-0.010	118.5	179.2	527.72	4.60
$NO_2PH_2\cdots IF$	3.279	2.018	0.004	1.834	-0.007	98.9	173.9	531.81	4.06
$RPH_2\cdots FI$	$R_{P\cdots F}$	R_{IF}	ΔR_{IF}	R_{RP}	ΔR_{RP}	Θ_{RPF}	Θ_{PFI}	$\Delta \nu_{IF}$	ΔE_{bind}
$CH_3PH_2\cdots FI$	3.144	2.015	0.001	1.856	0.000	161.8	105.2	543.10	3.52
$OHPH_2\cdots FI$	2.849	2.015	0.001	1.689	0.009	165.2	149.5	545.29	3.89
$CF_3PH_2\cdots FI$	2.900	2.016	0.002	1.876	-0.001	160.0	117.0	544.79	5.28
$CNPH_2\cdots FI$	2.856	2.016	0.002	1.798	0.006	161.4	120.5	545.88	5.82
$NO_2PH_2\cdots FI$	2.708	2.017	0.003	1.843	0.002	161.9	125.1	546.60	6.87

Table S1. Optimized dihalogen $RPH_2\cdots IF$ and $RPH_2\cdots FI$ complexes (MP2/mixed-PP level), showing key structural and energetic descriptors: bond lengths ($R_{P\cdots R}$, R_{IF}, ΔR_{IF}, R_{RP}, ΔR_{RP}; Å), orientation angles (Θ_{RPX}, Θ_{PXY}; degrees), dihalogen infrared frequency shift ($\Delta\nu_{IF}$; cm^{-1}) and binding energy (ΔE_{bind}; kcal mol^{-1}).

$RPH_2\cdots IF$	$R_{P\cdots I}$	R_{IF}	ΔR_{IF}	R_{RP}	ΔR_{RP}	Θ_{RPI}	Θ_{PIF}	$\Delta\nu_{IF}$	ΔE_{bind}
$CH_3PH_2\cdots IF$	2.960	2.066	0.059	1.895	0.023	117.0	179.1	470.56	14.64
$OHPH_2\cdots IF$	3.014	2.052	0.046	1.753	0.065	119.1	179.6	484.85	10.42
$CF_3PH_2\cdots IF$	3.092	2.034	0.027	1.925	0.029	122.1	174.0	505.67	6.31
$CNPH_2\cdots IF$	3.091	2.036	0.030	1.814	0.017	120.7	177.2	503.36	6.54
$NO_2PH_2\cdots IF$	3.102	2.031	0.025	1.933	0.053	122.9	175.0	509.02	5.05
**$RPH_2\cdots FI$**	**$R_{P\cdots F}$**	**R_{IF}**	**ΔR_{IF}**	**R_{RP}**	**ΔR_{RP}**	**Θ_{RPF}**	**Θ_{PFI}**	**$\Delta\nu_{IF}$**	**ΔE_{bind}**
$CH_3PH_2\cdots FI$	2.534	2.069	0.063	1.920	0.047	172.7	167.7	409.3	1.52
$OHPH_2\cdots FI$	2.372	2.083	0.077	1.782	0.094	173.9	162.7	386.6	3.81
$CF_3PH_2\cdots FI$	2.867	2.010	0.003	1.930	0.034	155.7	143.2	547.6	3.48
$CNPH_2\cdots FI$	2.998	2.012	0.006	1.836	0.039	180.0	174.3	548.1	3.38
$NO_2PH_2\cdots FI$	2.527	2.030	0.023	1.940	0.060	169.7	167.1	482.6	4.94

Table S2. Optimized dihalogen $RPH_2\cdots IF$ and $RPH_2\cdots FI$ complexes (B3LYP/LANL2DZ level), showing key structural and energetic descriptors: bond lengths ($R_{P\cdots X}$, R_{IF}, ΔR_{IF}, R_{RP}, ΔR_{RP}; Å), orientation angles (Θ_{RPX}, Θ_{PXY}; degrees), dihalogen infrared frequency shift ($\Delta\nu_{IF}$; cm^{-1}) and binding energy (ΔE_{bind}; kcal mol^{-1}).

**$RPH_2 \cdots IF$**	**$R_{P \cdots I}$**	**R_{IF}**	**ΔR_{IF}**	**R_{RP}**	**ΔR_{RP}**	**Θ_{RPI}**	**Θ_{PIF}**	**$\Delta \nu_{IF}$**	**ΔE_{bind}**
$CH_3PH_2 \cdots IF$	3.075	2.050	0.036	1.907	-0.017	116.7	179.0	497.05	10.07
$OHPH_2 \cdots IF$	3.195	2.035	0.021	1.771	-0.019	116.9	179.3	512.94	6.32
$CF_3PH_2 \cdots IF$	3.306	2.024	0.010	1.926	-0.004	120.0	177.4	526.68	3.87
$CNPH_2 \cdots IF$	3.262	2.028	0.014	1.834	-0.013	118.9	178.2	522.13	4.71
$NO_2PH_2 \cdots IF$	3.332	2.024	0.010	1.903	-0.006	117.3	177.6	527.13	3.28
**$RPH_2 \cdots FI$**	**$R_{P \cdots F}$**	**R_{IF}**	**ΔR_{IF}**	**R_{RP}**	**ΔR_{RP}**	**Θ_{RPF}**	**Θ_{PFI}**	**$\Delta \nu_{IF}$**	**ΔE_{bind}**
$CH_3PH_2 \cdots FI$	3.270	2.015	0.001	1.925	0.002	159.3	112.0	542.51	1.39
$OHPH_2 \cdots FI$	2.926	2.014	0.000	1.797	0.007	159.7	165.9	550.11	2.78
$CF_3PH_2 \cdots FI$	2.904	2.014	0.000	1.930	0.000	154.3	151.9	553.43	4.16
$CNPH_2 \cdots FI$	2.959	2.014	0.000	1.853	0.006	155.5	145.0	551.28	3.76
$NO_2PH_2 \cdots FI$	2.792	2.014	0.000	1.908	-0.001	157.6	146.0	555.79	4.97

Table S3. Optimized dihalogen $RPH_2 \cdots IF$ and $RPH_2 \cdots FI$ complexes (MP2/LANL2DZ level), showing key structural and energetic descriptors: bond lengths ($R_{P \cdots X}$, R_{IF}, ΔR_{IF}, R_{RP}, ΔR_{RP}; Å), orientation angles (Θ_{RPX}, Θ_{PXY}; degrees), dihalogen infrared frequency shift ($\Delta \nu_{IF}$; cm^{-1}) and binding energy (ΔE_{bind}; kcal mol^{-1}).

$RPH_2\cdots IF$	$R_{P\cdots I}$	R_{IF}	ΔR_{IF}	R_{PR}	ΔR_{PR}	Θ_{RPI}	Θ_{PIF}	$\Delta\nu_{IF}$	ΔE_{bind}
$CH_3PH_2\cdots IF$	2.840	2.073	0.066	1.844	-0.029	113.0	177.3	-86.59	-16.04
$OHPH_2\cdots IF$	2.835	2.068	0.061	1.650	-0.038	113.8	177.4	-82.02	-13.64
$CF_3PH_2\cdots IF$	2.975	2.039	0.032	1.895	-0.004	119.2	179.0	-51.73	-8.37
$CNPH_2\cdots IF$	3.013	2.035	0.028	1.783	-0.015	118.6	178.6	-46.66	-7.12
$NO_2PH_2\cdots IF$	3.006	2.031	0.024	1.879	-0.004	118.1	177.6	-43.23	-6.23
**$RPH_2\cdots FI$**	$R_{P\cdots F}$	R_{IF}	ΔR_{IF}	R_{PR}	ΔR_{PR}	Θ_{RPF}	Θ_{PFI}	$\Delta\nu_{IF}$	ΔE_{bind}
$CH_3PH_2\cdots FI$	2.250	2.131	0.124	1.874	0.001	166.0	162.2	-232.56	-4.74
$OHPH_2\cdots FI$	1.949	2.280	0.273	1.683	-0.006	162.2	154.1	-321.47	-7.70
$CF_3PH_2\cdots FI$	2.559	2.026	0.019	1.903	0.004	172.0	159.2	-59.98	-5.79
$CNPH_2\cdots FI$	2.608	2.020	0.013	1.807	0.009	171.7	156.9	-39.62	-6.20
$NO_2PH_2\cdots FI$	2.271	2.064	0.057	1.909	0.026	176.8	155.9	-129.23	-8.47

Table S4. Optimized dihalogen $RPH_2\cdots IF$ and $RPH_2\cdots FI$ complexes (B3LYP-D3/mixed-PP level), showing key structural and energetic descriptors: bond lengths ($R_{P\cdots X}$, R_{IF}, ΔR_{IF}, R_{RP}, ΔR_{RP}; Å), orientation angles (Θ_{RPX}, Θ_{PXY}; degrees), dihalogen infrared frequency shift ($\Delta\nu_{IF}$; cm^{-1}) and binding energy (ΔE_{bind}; kcal mol^{-1}).

	**B3LYP**/mixed-PP	**B3LYP**/LANL2DZ	**MP2**/mixed-PP	**MP2**/LANL2DZ	**B3LYP-D3** /mixed-PP
**CH_3**	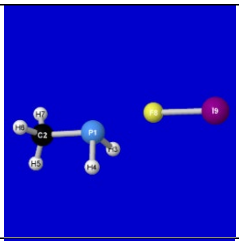	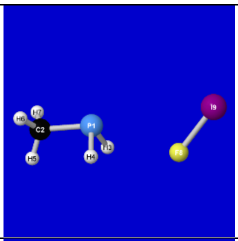	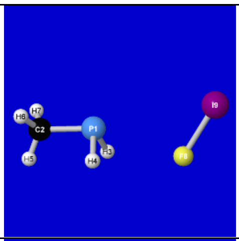	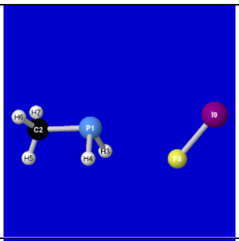	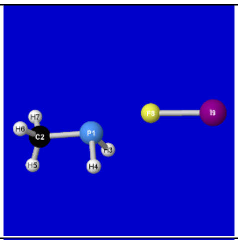
**OH**	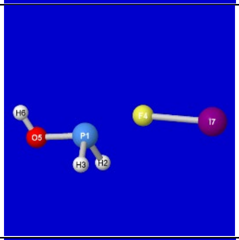	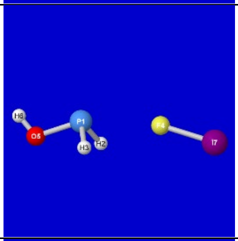	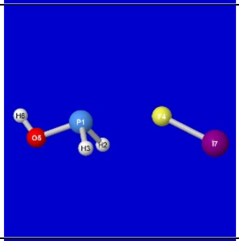	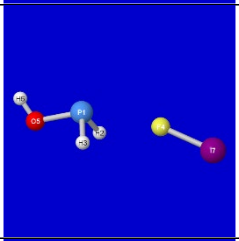	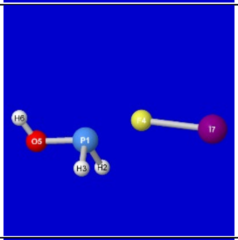
**NO_2**	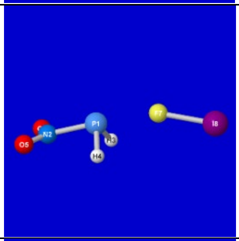	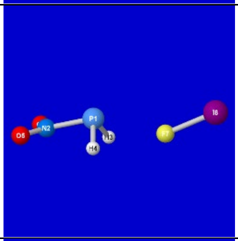	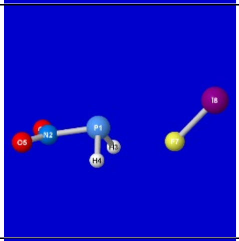	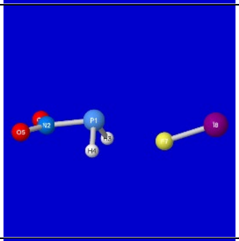	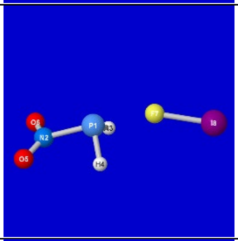
**CF_3**	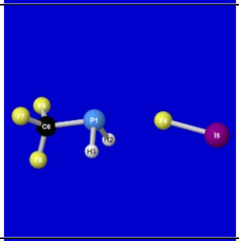	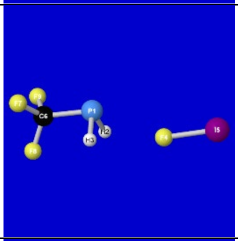	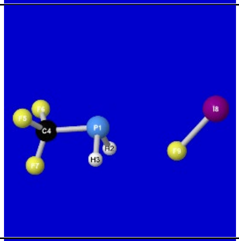	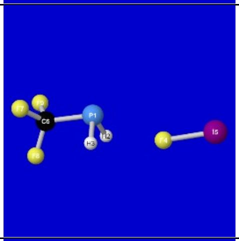	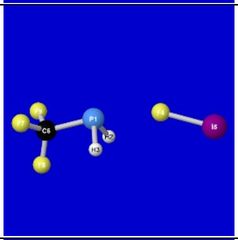
**CN**	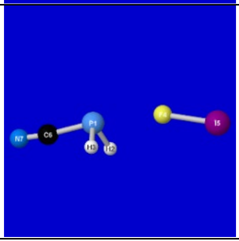	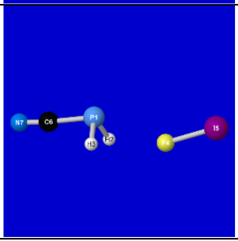	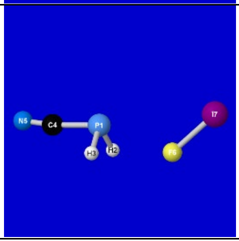	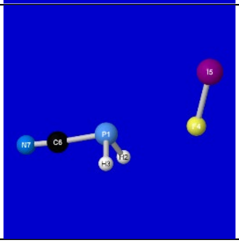	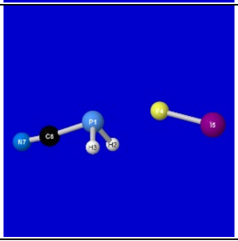

Figure S1. Optimized structures of $XH_2P\cdots FI$ complexes evaluated at B3LYP/mixed-PP, B3LYP/LANL2DZ, B3LYP/mixed-PP, MP2/LANL2DZ and B3LYP-D3/mixed-PP of theory.

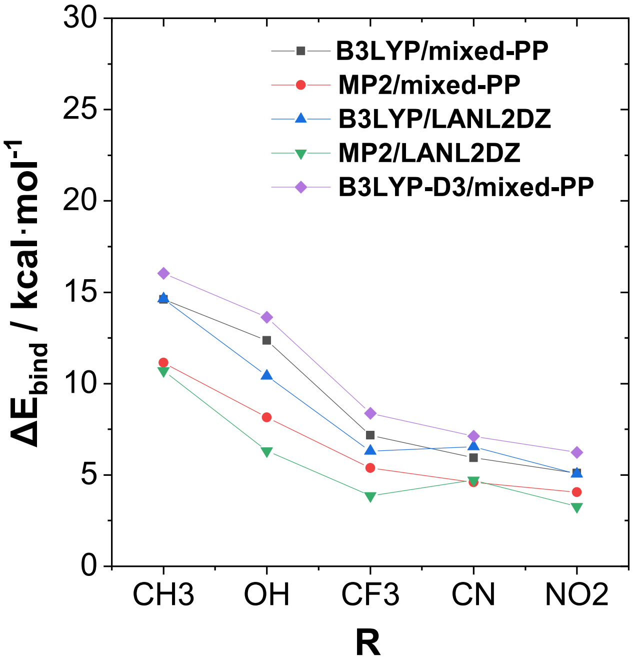

Figure S2. Calculated trend of ΔE_{bind} with substituent R in the $RPH_2\cdots IF$ complexes for five theoretical levels. Note the reversed ordering of cyano substituent effect on binding energy when diffuse functions are absent ("pure" LANL2DZ; see text).

**species**	$\Delta E^{(2)}_{n(P)\to\sigma^*(IF)}$	$\Delta E^{(2)}_{n(I)\to\sigma^*(P\underline{R})}$	$\Delta E^{(\$DEL)}_{n(P)\to\sigma^*(IF)}$	$\Delta E^{(\$DEL)}_{n(I)\to\sigma^*(P\underline{R})}$
$\underline{C}H_3PH_2$···IF	38.65	1.25	54.41	1.39
$\underline{O}HPH_2$···IF	37.75	2.02	54.64	2.47
$\underline{C}F_3PH_2$···IF	23.50	1.51	29.97	1.57
$\underline{C}NPH_2$···IF	21.78	1.31	26.08	1.60
$\underline{N}O_2PH_2$···IF	19.96	2.26	24.76	2.17

Table S5. $\Delta E_{n\sigma^*}$ stabilization energy estimates (kcal/mol) for halogen-type [n(P)→σ*(IF)] and leading pnicogen-type [n(F)→σ*(P$\underline{R}$)] interactions of RPH_2···IF complexes (B3LYP-D3/mixed-PP level), showing perturbative $\Delta E^{(2)}_{n\sigma^*}$ values in the first two columns and variational deletion $\Delta E^{(\$DEL)}_{n\sigma^*}$ values in the final two columns (The connected R-atom of each $\sigma^*_{P\underline{R}}$ acceptor NBO is shown underlined in the species listing.)

species	$b_{P\cdots I}$	b_{IF}	$b_{P\wedge F}$	$b_{P\cdots I} + b_{IF}$	$b_{P\cdots I} + b_{IF} + b_{P\wedge F}$
$CH_3PH_2\cdots IF$	0.49	0.59	0.00	1.07	1.07
$OHPH_2\cdots IF$	0.45	0.58	0.00	1.03	1.03
$CF_3PH_2\cdots IF$	0.31	0.63	0.11	0.94	1.05
$CNPH_2\cdots IF$	0.28	0.66	0.10	0.94	1.04
$NO_2PH_2\cdots IF$	0.32	0.66	0.10	0.98	1.08
**species**	$b_{P\cdots F}$	b_{IF}	$b_{P\wedge I}$	$b_{P\cdots F} + b_{IF}$	$b_{P\cdots F} + b_{IF} + b_{P\wedge I}$
$CH_3PH_2\cdots FI$	0.07	0.63	0.35	0.71	1.06
$OHPH_2\cdots FI$	0.38	0.41	0.39	0.79	1.17
$CF_3PH_2\cdots FI$	0.10	0.87	0.08	0.97	1.05
$CNPH_2\cdots FI$	0.09	0.91	0.05	1.00	1.05
$NO_2PH_2\cdots FI$	0.19	0.77	0.14	0.96	1.10

Table S6. NRT bond orders and bond order sums for $b_{P\cdots I}$ halogen bond, b_{IF} covalent bond, and $b_{P\wedge F}$ long bond of $RPH_2\cdots IF$ complexes (P···IF triad; upper rows), or $b_{P\cdots F}$ pnicogen bond, b_{IF} covalent bond, and $b_{P\wedge I}$ long bond of $RPH_2\cdots FI$ complexes (F···P$\underline{R}$ triad; lower rows) (B3LYP-D3/mixed-PP level). Note the near-constant "bond conservation" of the summed bond orders within each 3c/4e triad (final column).

Example of $CH_3PH_2\cdots IF$ complex

```
 %mem=4GB
%nprocshared=8
%chk=ch3if.chk
# opt b3lyp/genecp

E(RB3LYP) =  -493.718683170,
Vibrational temperatures:     87.39   128.19   186.81   254.15   259.71
          (Kelvin)            472.60   666.92  1003.13  1011.05  1074.17
                             1421.02  1485.51  1579.36  1934.10  2111.67
                             2112.53  3499.59   3521.26  4388.16  4505.73
                             4513.01

 Zero-point correction=                                  0.057345 (Hartree/Particle)
 Thermal correction to Energy=                     0.064691
 Thermal correction to Enthalpy=                  0.065635
 Thermal correction to Gibbs Free Energy=          0.023712
 Sum of electronic and zero-point Energies=       -493.661338
 Sum of electronic and thermal Energies=           -493.653992
 Sum of electronic and thermal Enthalpies=        -493.653048
 Sum of electronic and thermal Free Energies=    -493.694972


0 1
 P                  2.06518300   -0.53194900    0.00000600
 H                  2.60198300   -1.28230400    1.07132900
 H                  2.60315900   -1.28304500   -1.07021200
 I                 -0.75267600   -0.04725500   -0.00004800
 C                  3.12840900    0.97637500    0.00002000
 F                 -2.78158100    0.36821900    0.00015100
 H                  2.88938900    1.57261600    0.88173000
 H                  2.89209500    1.57059700   -0.88379200
 H                  4.19120800    0.73364500    0.00191400


P H C 0
6-311++g(d,p)
****
F I 0
lanl2dz
****

F I 0
lanl2dz
```

OPT input file

```
%mem=4GB
%nprocshared=8
%chk=ch3if.chk
# opt b3lyp/genecp

ch3if

0 1
 P                 -1.89690040   -0.53914345   -0.00004943
 H                 -2.43848173   -1.27535423   -1.07857229
 H                 -2.43753737   -1.27491560    1.07928076
 I                  0.67663393   -0.16911518    0.00034032
 C                 -2.87585411    0.99514780   -0.00000296
 F                  2.55893899    0.15496423   -0.00010186
 H                 -2.64285494    1.56692240   -0.87389380
 H                 -2.64210512    1.56734790    0.87340900
 H                 -3.91814079    0.75320106    0.00050324
 X                  0.50700835    0.81629722   -0.01342806

P H C 0
6-311++g(d,p)
****
F I 0
lanl2dz
****

F I 0
lanl2dz
```

NBO input file

```
%mem=4GB
%nprocshared=8
%chk=ch3if_nbo.chk
# b3lyp/genecp pop=nbo6read

ch3if_nbo

0 1
 P                  2.06518300   -0.53194900    0.00000600
 H                  2.60198300   -1.28230400    1.07132900
 H                  2.60315900   -1.28304500   -1.07021200
 I                 -0.75267600   -0.04725500   -0.00004800
 C                  3.12840900    0.97637500    0.00002000
 F                 -2.78158100    0.36821900    0.00015100
 H                  2.88938900    1.57261600    0.88173000
 H                  2.89209500    1.57059700   -0.88379200
 H                  4.19120800    0.73364500    0.00191400

P H C 0
6-311++g(d,p)
****
F I 0
lanl2dz
****

F I 0
lanl2dz

$nbo file= ch3if_nbo archive $end
```

STERIC input file

```
%mem=4GB
%nprocshared=8
%chk=ch3if_steric.chk
# b3lyp/genecp pop=nbo6read

ch3if_steric

0 1
 P                  2.06518300   -0.53194900    0.00000600
 H                  2.60198300   -1.28230400    1.07132900
 H                  2.60315900   -1.28304500   -1.07021200
 I                 -0.75267600   -0.04725500   -0.00004800
 C                  3.12840900    0.97637500    0.00002000
 F                 -2.78158100    0.36821900    0.00015100
 H                  2.88938900    1.57261600    0.88173000
 H                  2.89209500    1.57059700   -0.88379200
 H                  4.19120800    0.73364500    0.00191400

P H C 0
6-311++g(d,p)
****
F I 0
lanl2dz
****

F I 0
lanl2dz

$NBO STERIC <8 18> FILE=ch3if_steric $END
```

Deletion input file

```
%mem=4GB
%nprocshared=8
%chk=ch3if_deletion.chk
# b3lyp/genecp pop=nbo6del

ch3if_deletion

0 1
 P                 2.06518300   -0.53194900    0.00000600
 H                 2.60198300   -1.28230400    1.07132900
 H                 2.60315900   -1.28304500   -1.07021200
 I                -0.75267600   -0.04725500   -0.00004800
 C                 3.12840900    0.97637500    0.00002000
 F                -2.78158100    0.36821900    0.00015100
 H                 2.88938900    1.57261600    0.88173000
 H                 2.89209500    1.57059700   -0.88379200
 H                 4.19120800    0.73364500    0.00191400

P H C 0
6-311++g(d,p)
****
F I 0
lanl2dz
****

F I 0
lanl2dz

$nbo file=ch3if_deletion archive $end
$del
delete 1 element 8 25
delete 1 element 11 24
ZERO 2 DELOC FROM 1 TO 2  FROM 2 TO 1
$end
```

Calculated coordinates of all complexes

1. $CH_3PH_2\cdots IF$

0 1

P	2.06518300	-0.53194900	0.00000600
H	2.60198300	-1.28230400	1.07132900
H	2.60315900	-1.28304500	-1.07021200
I	-0.75267600	-0.04725500	-0.00004800
C	3.12840900	0.97637500	0.00002000
F	-2.78158100	0.36821900	0.00015100
H	2.88938900	1.57261600	0.88173000
H	2.89209500	1.57059700	-0.88379200
H	4.19120800	0.73364500	0.00191400

2. $OHPH_2\cdots IF$

0 1

P	-2.09333600	-0.45445300	-0.00000700
H	-2.65789700	-1.19246100	-1.06735600
H	-2.65754000	-1.19227300	1.06765900
I	0.73070800	-0.04170800	-0.00001200
F	2.77023600	0.28533600	0.00003900
O	-3.05045300	0.89182100	0.00002400
H	-2.54054400	1.70944900	-0.00011000

3. $CF_3PH_2\cdots IF$

0 1

P	-1.20256400	0.98516300	-0.00005200
H	-1.65751100	1.79154900	1.06852300
H	-1.65750200	1.79148200	-1.06867700
I	1.63678500	0.04250900	-0.00000200
F	3.59347700	-0.52662000	0.00002100
C	-2.66348200	-0.22035400	-0.00001400
F	-2.61140800	-1.00820400	-1.08938400
F	-2.61231900	-1.00692500	1.09032000
F	-3.86033000	0.39826700	-0.00083500

4. $CNPH_2\cdots IF$

0 1

P	1.90965300	0.81304700	0.00006000
H	2.38905700	1.60696000	-1.06765000
H	2.38625800	1.60419100	1.07106600
I	-1.01103400	0.00159500	-0.00022800
F	-2.99142900	-0.46005200	0.00078400
C	3.20738500	-0.40947500	0.00017800
N	3.97760600	-1.27058100	-0.00005100

5. $NO_2PH_2\cdots IF$

0 1

P	1.52504700	0.90302300	0.01294300
H	1.99518400	1.71582600	-1.04265000
H	1.98876700	1.67839200	1.09907700
N	2.97854200	-0.28963000	-0.00345100
O	3.38174000	-0.63664400	-1.09667700
O	3.37288100	-0.67690400	1.07943200
I	-1.35368400	-0.00544200	-0.00077700
F	-3.33346600	-0.45725900	-0.00525500

6. $CH_3PH_2\cdots FI$

0 1

P	-2.68274200	0.06560500	-0.00019700
C	-4.54641600	-0.12138100	0.00033100
H	-2.55106800	1.00114100	1.05459600
H	-2.55165700	1.00238400	-1.05396500
H	-5.06520600	0.83947200	0.00099100
H	-4.84731100	-0.68873000	-0.88221200
H	-4.84670200	-0.68960500	0.88251800
F	-0.38265700	-0.26290500	-0.00051500
I	1.71368800	0.01218300	0.00006900

7. $OHPH_2\cdots FI$

0 1

P	-2.53886100	0.08094200	-0.02457200
H	-2.38215900	1.08885800	0.94451400
H	-2.37749100	0.87141300	-1.18712900
F	-0.56680600	-0.37447300	0.03729900
O	-4.21811000	-0.00367700	0.06299700
H	-4.57039500	-0.86272300	-0.19034600
I	1.62753000	0.02052800	-0.00071900

8. $CF_3PH_2\cdots FI$

0 1

P	1.71714200	-0.27184700	-0.00114700
H	1.48778000	0.65792900	-1.04675600
H	1.48523300	0.66305100	1.03926900
F	-0.90414500	-0.38225500	0.00151900
I	-2.88076300	0.05654900	-0.00007800
C	3.59002800	0.04709400	0.00009700
F	4.16443400	-0.51170100	1.09220200
F	3.95171000	1.35289200	-0.00265000
F	4.16690200	-0.51704200	-1.08793100

9. $CNPH_2\cdots FI$

0 1

P	2.59027500	0.20761700	-0.00595700
H	2.30827400	-0.72855100	1.02096300
H	2.32191700	-0.69392500	-1.06723200
F	-0.05784600	0.36469900	0.00989900
I	-2.03406500	-0.05515000	-0.00026400
C	4.36648100	-0.11462800	0.00066600
N	5.52040700	-0.19476500	0.00807500

10. $NO_2PH_2\cdots FI$

0 1

P	1.94193000	0.00006900	0.21230100
N	3.82248800	-0.00002300	-0.07291200
H	1.74685700	-1.05106800	-0.71285300
H	1.74696700	1.05121200	-0.71287200
O	4.37774100	1.08576900	-0.11737400
O	4.37763100	-1.08587200	-0.11733600
F	-0.38507100	0.00002500	0.44483000
I	-2.37655700	-0.00000800	-0.06366400

11. $CH_3PH_2\cdots Cl_2$

0 1

P	-1.84783000	-0.53208800	-0.00004200
H	-2.38764700	-1.26717300	-1.08021600
H	-2.39023000	-1.26898500	1.07763500
C	-2.83792900	1.01967100	0.00000500
H	-3.91019500	0.81959600	-0.00000800
H	-2.57532400	1.60214900	0.88405000
H	-2.57530500	1.60209100	-0.88407100
Cl	0.60136700	-0.18207400	0.00035800
Cl	2.84473400	0.20417000	-0.00016900

12. $OHPH_2\cdots Cl_2$

0 1

P	-1.92919100	-0.46296200	0.00000900
H	-2.60026500	-1.09562600	-1.07521700
H	-2.59928800	-1.09517800	1.07607800
Cl	0.57321700	-0.21786700	-0.00016100
Cl	2.78706700	0.16972500	0.00008800
O	-2.62650800	1.02687900	0.00013100
H	-1.97536500	1.73862500	-0.00080800

13. $CF_3PH_2\cdots Cl_2$

0 1

P	-0.85217000	0.96295200	-0.00013100
H	-1.33561000	1.77609200	1.05512800
H	-1.33610000	1.77652600	-1.05483200
Cl	1.99574600	0.16639700	0.00020400
Cl	4.04388600	-0.29667700	-0.00012700
C	-2.33674400	-0.21540000	-0.00001400
F	-2.29961400	-1.00934200	1.08962800
F	-2.29987800	-1.00924900	-1.08973100
F	-3.53373100	0.40862000	0.00015300

14. $CNPH_2\cdots Cl_2$

0 1

P	-1.72524700	-0.76872800	0.00002900
H	-2.21544600	-1.57796100	-1.05567300
H	-2.21544500	-1.57786900	1.05580300
Cl	1.20080000	-0.07793500	0.00008800
Cl	3.26116900	0.28657100	-0.00008400
C	-3.07657300	0.40393600	-0.00002300
N	-3.86920600	1.24519100	-0.00007000

15. $NO_2PH_2\cdots Cl_2$

0 1

P	-1.24028600	0.77126000	0.11141500
H	-1.66236700	1.45498300	1.27729200
H	-1.66387300	1.75854600	-0.81096200
Cl	1.72195300	0.01806400	0.00167600
Cl	3.78737600	-0.24352600	-0.03467900
N	-2.81388400	-0.24820600	-0.03564500
O	-3.25116600	-0.73211000	0.99288600
O	-3.25269400	-0.41940500	-1.15876100

16. $CH_3PH_2\cdots ClF$

```
0 1
 P                 -1.22566500   -0.54515600   -0.00000400
 H                 -1.68896200   -1.31691300   -1.08731400
 H                 -1.68907900   -1.31703000    1.08717400
 C                 -2.30131000    0.93838700    0.00000100
 H                 -3.35817200    0.66761500   -0.00004900
 H                 -2.07598100    1.53499800    0.88493700
 H                 -2.07589600    1.53503200   -0.88489300
 F                  2.87333700    0.33677900   -0.00002200
 Cl                 1.01299400   -0.09339400    0.00002300
```

17. $OHPH_2\cdots ClF$

```
0 1
 P                 -1.27101000   -0.47036100    0.00002800
 H                 -1.80611200   -1.18630300   -1.09332500
 H                 -1.80611200   -1.18609700    1.09352000
 F                  2.81853100    0.27127900    0.00001600
 Cl                 0.94796700   -0.11464600    0.00002500
 O                 -2.14914500    0.90364500   -0.00014300
 H                 -1.61168000    1.70612500   -0.00003800
```

18. $CF_3PH_2\cdots ClF$

```
0 1
 P                 -0.20755300    0.94696900   -0.00003800
 H                 -0.60072500    1.77305500    1.07670700
 H                 -0.60142400    1.77395200   -1.07584300
 F                  3.85200900   -0.48815100    0.00011300
 Cl                 2.15227900    0.06641400   -0.00010700
 C                 -1.71911800   -0.19582500   -0.00000200
 F                 -1.70419600   -0.98224600   -1.08914500
 F                 -1.70480900   -0.98130100    1.08982100
 F                 -2.88285000    0.48440700   -0.00061800
```

19. $CNPH_2\cdots ClF$

```
0 1
 P                 -0.96895800   -0.79275800    0.00000300
 H                 -1.37778200   -1.61259800   -1.07595600
 H                 -1.37733200   -1.61212100    1.07649600
 F                  3.16592200    0.50345200    0.00009500
 Cl                 1.46028200    0.01644100   -0.00009000
 C                 -2.33207100    0.35222700    0.00002200
 N                 -3.14802600    1.17030800   -0.00000700
```

20. $NO_2PH_2\cdots ClF$

```
0 1
 H                 -0.86469700    1.65433300   -1.04154000
 P                 -0.51526000    0.78921700    0.01831300
 H                 -0.86618500    1.60592800    1.11542500
 N                 -2.09972300   -0.22318100   -0.00522400
 O                 -2.53926000   -0.51309300   -1.10085000
 O                 -2.53844000   -0.56416300    1.07597300
 Cl                 1.89492900   -0.05315000   -0.00091400
 F                  3.61840600   -0.44607200   -0.01082900
```

21. $CH_3PH_2\cdots ClBr$

0 1

P	2.66289200	-0.52804800	-0.00000300
H	3.26575100	-1.23573800	1.06833200
H	3.26568700	-1.23556900	-1.06848700
C	3.61588700	1.05592200	0.00001600
H	4.69397000	0.89111300	0.00147000
H	3.33793000	1.63166700	-0.88389600
H	3.33572900	1.63302700	0.88234200
Br	-2.28826000	0.10858000	-0.00000500
Cl	0.03243200	-0.22938800	0.00002300

22. $OHPH_2\cdots ClBr$

0 1

H	-3.50546000	-1.01966500	1.06226900
P	-2.74918600	-0.45951100	-0.00002800
O	-3.32217400	1.09429800	-0.00008400
H	-2.60505200	1.73824700	0.00061300
H	-3.50483600	-1.01937300	-1.06290400
Br	2.23731400	0.09281200	-0.00004100
Cl	-0.05149900	-0.28290300	0.00015100

23. $CF_3PH_2\cdots ClBr$

0 1

P	1.71756200	0.97233800	-0.00024200
H	2.22377500	1.76548400	-1.06161500
H	2.24023100	1.78556600	1.03774800
C	3.18310400	-0.22967800	-0.00004500
Br	-3.50772100	-0.16378600	-0.00068100
Cl	-1.32228400	0.24218100	0.00257600
F	3.14300200	-1.01195100	1.09993500
F	3.12156800	-1.03828300	-1.07991900
F	4.39354100	0.36771800	-0.01914700

24. $CNPH_2\cdots ClBr$

0 1

H	-3.10964200	1.55081600	-1.06070900
P	-2.61343300	0.74793200	-0.00139500
H	-3.12186700	1.56543300	1.04084200
C	-3.97278000	-0.41926600	-0.00118200
N	-4.76899000	-1.25732400	-0.00017700
Br	2.69358700	-0.14864700	-0.00210400
Cl	0.49276900	0.12848700	0.00722100

25. $NO_2PH_2\cdots ClBr$

0 1

H	-2.53188700	1.03480900	1.60761900
P	-2.12172400	-0.00800400	0.74076400
H	-2.53319400	-1.06732700	1.58673000
N	-3.72356200	0.00280200	-0.24583400
O	-4.17047600	1.09308600	-0.55591500
O	-4.17192500	-1.08054200	-0.57740400
Br	3.21583600	0.00139400	-0.11665600
Cl	1.00828300	-0.00095200	0.03320700

26. $CH_3PH_2\cdots ClI$

```
0 1
 P                  3.30569100   -0.52631000   -0.00027200
 H                  3.98814800   -1.16173400    1.06687600
 H                  3.97926800   -1.15749100   -1.07545800
 Cl                 0.64111400   -0.30829300    0.00135000
 I                 -1.98893300    0.07527600   -0.00020400
 C                  4.06315800    1.15758400   -0.00000300
 H                  3.71047400    1.69835300   -0.87937400
 H                  3.71884900    1.69429700    0.88516000
 H                  5.15346400    1.12708300   -0.00522000
```

27. $OHPH_2\cdots ClI$

```
0 1
 P                  3.25135600   -0.34411000   -0.00019700
 H                  3.51039300   -1.24735900    1.06081600
 H                  3.51056200   -1.24776600   -1.06100500
 Cl                 0.60373200    0.06693200   -0.00042800
 I                 -2.03602800    0.01504300    0.00009700
 O                  4.66775600    0.53015800    0.00119700
 H                  4.51268000    1.48038800   -0.00429100
```

28. $CF_3PH_2\cdots ClI$

```
0 1
 P                 -2.18699000    0.51837700    0.00007800
 H                 -2.38421100    1.44536000    1.05337000
 H                 -2.38342600    1.44305800   -1.05537300
 Cl                 0.64578400   -0.01850400   -0.00040800
 I                  3.21407800   -0.03507300    0.00009100
 C                 -3.97933800   -0.10946600    0.00008200
 F                 -4.20692300   -0.87082100    1.09033500
 F                 -4.20624500   -0.87269000   -1.08902900
 F                 -4.90638300    0.87308600   -0.00103400
```

29. $CNPH_2\cdots ClI$

```
0 1
 P                 -3.07618400   -0.47048300   -0.00001800
 H                 -3.28794000   -1.40552500   -1.04353100
 H                 -3.27433900   -1.37755000    1.07042500
 Cl                -0.20602400    0.05264700   -0.00691200
 I                  2.35624200    0.04545900    0.00130600
 C                 -4.73475000    0.20675500    0.00170800
 N                 -5.75212500    0.75649600    0.00163300
```

30. $NO_2PH_2\cdots ClI$

```
0 1
 H                 -2.48336900    1.05327800    1.10893400
 P                 -2.49960300   -0.00061600    0.16554100
 H                 -2.48340000   -1.05828800    1.10470900
 N                 -4.38890300    0.00008900    0.00460000
 O                 -4.93146100    1.08739300   -0.07666500
 O                 -4.93270000   -1.08650300   -0.07748700
 Cl                 0.25571500    0.00061800   -0.36028900
 I                  2.78772200   -0.00007600    0.04960700
```

31. $CH_3PH_2\cdots Br_2$

0 1

H	-3.31274100	-1.19468000	1.07172100
P	-2.73382500	-0.47475400	0.00002400
H	-3.31287000	-1.19478000	-1.07153800
C	-3.70802800	1.09313600	0.00000100
H	-4.78297500	0.90906700	-0.00002700
H	-3.43818600	1.67343800	0.88338500
H	-3.43814000	1.67342800	-0.88337800
Br	-0.06661100	-0.18463200	-0.00003100
Br	2.39633900	0.14737600	0.00001600

32. $OHPH_2\cdots Br_2$

0 1

H	3.43367100	-1.06277100	-1.06832400
P	2.77574900	-0.40417600	0.00000000
O	3.54585200	1.05624000	-0.00000800
H	2.92838500	1.79658200	0.00004400
H	3.43373500	-1.06280400	1.06826700
Br	0.08564900	-0.18608900	0.00000400
Br	-2.36561600	0.12728100	-0.00000200

33. $CF_3PH_2\cdots Br_2$

0 1

H	2.42685600	1.76294600	1.05592000
P	1.91593200	0.96901200	-0.00027900
H	2.42636000	1.76205200	-1.05738700
C	3.35014900	-0.27054200	-0.00003700
F	3.28142100	-1.05979800	1.09065900
F	3.27955600	-1.06245900	-1.08866800
F	4.56990700	0.30674700	-0.00179900
Br	-1.03190100	0.22967900	0.00027700
Br	-3.36441400	-0.23246000	-0.00015900

34. $CNPH_2\cdots Br_2$

0 1

H	3.24628300	-1.53644200	-1.05738200
P	2.71147200	-0.75930500	0.00008200
H	3.24664600	-1.53647100	1.05732400
C	3.98303800	0.49808700	-0.00000900
N	4.72132500	1.38733600	0.00001200
Br	-0.31489500	-0.14874100	-0.00008400
Br	-2.65974800	0.19910100	0.00005000

35. $NO_2PH_2\cdots Br_2$

0 1

H	2.66800800	-1.01862100	1.61959200
P	2.26621500	0.01859700	0.74448900
H	2.66949900	1.09629900	1.56849500
N	3.85647200	-0.00690000	-0.26156900
O	4.29831600	-1.10207300	-0.55775200
O	4.30054300	1.07200300	-0.60957400
Br	-0.75113500	0.00180900	0.04229300
Br	-3.10934800	-0.00374600	-0.13331600

36. CH_3PH_2···BrF

0 1

H	-2.06680200	-1.30340400	1.08186100
P	-1.59081100	-0.53049200	-0.00000800
H	-2.06670000	-1.30331300	-1.08198400
C	-2.69007200	0.94072300	0.00000100
H	-3.74336400	0.65689200	-0.00012900
H	-2.47299600	1.54104100	0.88449800
H	-2.47282500	1.54116500	-0.88437000
Br	0.80716900	-0.05934300	0.00001500
F	2.73048400	0.36196500	-0.00003100

37. $OHPH_2$···BrF

0 1

H	2.14230700	-1.18990600	-1.08551100
P	1.61516700	-0.45561900	0.00001000
O	2.55326000	0.88643400	-0.00004800
H	2.04481600	1.70691700	0.00027900
H	2.14223000	-1.18988800	1.08559000
Br	-0.76320700	-0.06411600	-0.00000400
F	-2.69674500	0.29553000	0.00000100

38. CF_3PH_2···BrF

0 1

H	1.04507400	1.77098300	1.08017000
P	0.65098400	0.95086300	0.00005600
H	1.04523400	1.77128700	-1.07977300
C	2.15136100	-0.20839900	0.00001300
F	2.13044200	-0.99304400	1.08910100
F	3.31707400	0.46748800	0.00051800
F	2.13097800	-0.99219500	-1.08969500
Br	-1.74210300	0.05553700	-0.00003200
F	-3.55512100	-0.53765000	0.00005300

39. $CNPH_2$··· BrF

0 1

H	-1.77269400	1.60975200	-1.08039200
P	-1.36176400	0.79807300	-0.00010800
H	-1.77379200	1.61067300	1.07908000
C	-2.70625600	-0.36817500	-0.00019700
N	-3.51246700	-1.19565000	0.00006100
Br	1.09847700	-0.00222500	0.00024800
F	2.92789600	-0.50389200	-0.00055400

40. NO_2PH_2···BrF

0 1

H	1.30300300	1.64204500	1.09681200
P	0.94027900	0.82511500	0.00509900
H	1.30382300	1.65640100	-1.07545600
N	2.49378400	-0.24000600	-0.00140300
O	2.92161400	-0.57394200	1.08568600
O	2.92250000	-0.55933200	-1.09253000
Br	-1.46095300	-0.02424000	-0.00026000
F	-3.30967500	-0.45339200	-0.00268700

41. $CH_3PH_2\cdots BrCl$

0 1

H	-2.61187800	-1.23978600	1.07682400
P	-2.07705900	-0.49646900	0.00000000
H	-2.61197700	-1.23985500	-1.07672900
C	-3.09823900	1.03626500	0.00000000
H	-4.16595300	0.81360700	0.00005000
H	-2.84792700	1.62441000	0.88385800
H	-2.84800000	1.62436000	-0.88391000
Br	0.48062900	-0.13360800	-0.00000400
Cl	2.82406100	0.25429400	0.00000400

42. $OHPH_2\cdots BrCl$

0 1

H	2.71447400	-1.12085000	-1.07590200
P	2.11395900	-0.42494100	0.00005000
O	2.95809200	0.98642400	-0.00001700
H	2.38741500	1.76410600	0.00009700
H	2.71422900	-1.12069800	1.07627500
Br	-0.45051800	-0.13310600	-0.00006400
Cl	-2.78953500	0.21287500	0.00006700

43. $CF_3PH_2\cdots BrCl$

0 1

H	1.67487100	1.77410200	1.06393100
P	1.19950200	0.97027800	0.00044100
H	1.67465200	1.77475100	-1.06264700
C	2.65306900	-0.24620400	0.00004000
F	2.59763600	-1.03445200	1.09023600
F	3.85842200	0.35846700	-0.00144700
F	2.59584100	-1.03598500	-1.08892000
Br	-1.56639400	0.15478500	-0.00046000
Cl	-3.75904500	-0.39032500	0.00053700

44. $CNPH_2\cdots BrCl$

0 1

H	2.48631300	-1.56815600	-1.06347300
P	1.98179300	-0.78136400	-0.00011300
H	2.48569300	-1.56821400	1.06350800
C	3.26845100	0.45660300	0.00015500
N	4.02253500	1.33230900	-0.00007600
Br	-0.86469700	-0.08902600	0.00008900
Cl	-3.07076300	0.34746900	-0.00010900

45. $NO_2PH_2\cdots BrCl$

0 1

H	1.95782900	1.62815000	1.06394400
P	1.54161400	0.79550500	-0.00070200
H	1.95983300	1.62845000	-1.06433700
N	3.09242100	-0.27036000	0.00041000
O	3.52267400	-0.60465200	1.08835400
O	3.52203500	-0.60719900	-1.08698400
Br	-1.29440900	0.03306100	-0.00056900
Cl	-3.51424600	-0.27994100	0.00099900

46. $CH_3PH_2\cdots BrI$

0 1

H	-3.97430700	-1.14628800	1.07069800
P	-3.35669900	-0.45592600	0.00002100
H	-3.97465400	-1.14663900	-1.07023400
C	-4.25874800	1.15602200	-0.00001300
H	-5.34146800	1.02454500	-0.00085200
H	-3.96205000	1.72283200	0.88351800
H	-3.96076000	1.72357000	-0.88262900
Br	-0.57446600	-0.22178000	-0.00004000
I	2.21174600	0.10352900	0.00001300

47. $OHPH_2\cdots BrI$

0 1

H	4.15520700	-0.94801400	-1.06530200
P	3.41400600	-0.37546200	0.00006200
O	4.00686200	1.16833400	-0.00002000
H	3.30075400	1.82458500	0.00001900
H	4.15491200	-0.94784700	1.06572700
Br	0.59033200	-0.25073300	-0.00006900
I	-2.17995200	0.09683400	0.00002300

48. $CF_3PH_2\cdots BrI$

0 1

H	-2.96355500	1.71926700	-1.06542400
P	-2.48262100	0.91767400	-0.00091900
H	-2.97106900	1.72976600	1.05218500
C	-3.95167700	-0.28127500	-0.00014000
F	-3.90095100	-1.07836000	-1.08557900
F	-3.90883600	-1.06721400	1.09375500
F	-5.15411300	0.33192600	-0.00760400
Br	0.46124800	0.26249700	0.00136300
I	3.15878100	-0.15832200	-0.00047100

49. $CNPH_2\cdots BrI$

0 1

H	3.76021400	-1.50186600	-1.06353400
P	3.25088200	-0.71497800	-0.00089600
H	3.76284000	-1.50540700	1.05786100
C	4.55119600	0.51116600	-0.00048400
N	5.30811000	1.38480500	-0.00005900
Br	0.25774800	-0.17798400	0.00139900
I	-2.44851700	0.13586300	-0.00050100

50. $NO_2PH_2\cdots BrI$

0 1

H	-2.87263400	1.05389500	1.36761100
P	-2.72995700	-0.00271600	0.43790400
H	-2.87538800	-1.06380300	1.36209300
N	-4.55069800	0.00102700	-0.07267900
O	-5.06519700	1.08955500	-0.25141100
O	-5.06875700	-1.08516400	-0.25529100
Br	0.17793200	-0.00013700	-0.17491200
I	2.89426900	0.00024800	0.02615200

51. $CH_3PH_2\cdots I_2$

0 1

P	3.47989600	-0.41096700	-0.00009200
H	4.09181900	-1.10819100	1.06863500
H	4.09214700	-1.10824000	-1.06860000
I	0.53273600	-0.18627100	0.00004800
I	-2.42877600	0.12336700	-0.00002500
C	4.40072500	1.19111000	0.00000400
H	4.11117200	1.76204900	-0.88320000
H	4.11098700	1.76204200	0.88315300
H	5.48117900	1.04409600	0.00011900

52. $OHPH_2\cdots I_2$

0 1

P	-3.53151400	-0.33884400	0.00017900
H	-4.22103200	-0.97273300	-1.06384200
H	-4.22088800	-0.97278800	1.06423100
I	-0.53981600	-0.18819600	0.00000500
I	2.40908900	0.11213600	-0.00012400
O	-4.25530700	1.14886500	0.00045400
H	-3.61437400	1.86845400	-0.00038900

53. $CF_3PH_2\cdots I_2$

0 1

P	2.73642500	-0.96182200	-0.00039600
H	3.28134500	-1.72935000	1.05772700
H	3.27954900	-1.72685900	-1.06123600
I	-0.37836000	-0.28028700	0.00032700
I	-3.24689200	0.21816900	-0.00017200
C	4.10103100	0.35428300	-0.00003900
F	3.98800700	1.13898000	-1.08917000
F	5.34815100	-0.15990900	-0.00082400
F	3.98882700	1.13760400	1.09015600

54. $CNPH_2\cdots I_2$

0 1

P	-3.44484300	-0.71408200	0.00009300
H	-4.00818700	-1.46542700	-1.06048500
H	-4.00818800	-1.46518600	1.06084100
C	-4.65062800	0.60416000	-0.00005700
N	-5.34486600	1.52819800	-0.00016200
I	-0.26316200	-0.17771900	0.00007500
I	2.62178200	0.16488000	-0.00008000

55. $NO_2PH_2\cdots I_2$

0 1

P	3.01170300	-0.01364300	-0.67487300
H	3.35553300	-1.08561900	-1.53169700
H	3.34669200	1.03499100	-1.56347600
N	4.66753500	0.00638000	0.22723600
O	5.13306300	-1.07500800	0.53503300
O	5.12639300	1.09980800	0.50031500
I	-3.02291400	0.00241400	0.05623600
I	-0.12097600	-0.00218400	0.00687400

56. $CH_3PH_2\cdots ICl$

0 1

P	2.41367500	-0.47202700	0.00002800
H	2.97149000	-1.20324500	1.07347400
H	2.97157900	-1.20336900	-1.07328800
C	3.40881600	1.08034400	-0.00000300
H	4.48039700	0.87877200	-0.00014700
H	3.14719900	1.66446800	-0.88324300
H	3.14741900	1.66435200	0.88338000
Cl	-3.01195600	0.28967800	0.00003600
I	-0.41835600	-0.11560700	-0.00002200

57. $OHPH_2\cdots ICl$

0 1

P	2.44959100	-0.40330400	0.00000400
H	3.05023900	-1.10703100	1.07092300
H	3.05017600	-1.10701300	-1.07096000
Cl	-2.99441700	0.23699400	0.00000300
I	-0.40094800	-0.10437600	-0.00000100
O	3.31865400	0.99776000	0.00000100
H	2.76180900	1.78457400	-0.00001300

58. $CF_3PH_2\cdots ICl$

0 1

P	-1.57223700	0.97756400	0.00004100
H	-2.05488800	1.76836400	1.06858200
H	-2.05521400	1.76888400	-1.06796800
Cl	3.83227400	-0.45846200	0.00006900
I	1.33400200	0.14772300	-0.00004500
C	-2.98899900	-0.28152400	0.00000700
F	-2.90893600	-1.06571200	1.08993500
F	-2.90835200	-1.06634400	-1.08941100
F	-4.20750100	0.29349400	-0.00053100

59. $CNPH_2\cdots ICl$

0 1

P	2.29454200	-0.77093000	-0.00001400
H	2.80414400	-1.54456600	1.06899600
H	2.80405400	-1.54445900	-1.06914200
Cl	-3.21176900	0.37501000	-0.00002700
I	-0.68985600	-0.08569200	0.00001600
C	3.54041600	0.50386900	-0.00000400
N	4.27051300	1.39946300	-0.00000100

60. $NO_2PH_2\cdots ICl$

0 1

P	1.87983100	-0.80036900	0.04491100
H	2.29051900	-1.56010000	1.16353400
H	2.29814900	-1.68844900	-0.97172400
N	3.41586600	0.28960100	-0.01514800
O	3.83460200	0.69353500	1.05218300
O	3.84375000	0.56099400	-1.12014800
I	-1.07021400	-0.02898200	0.00038300
Cl	-3.61192100	0.27804100	-0.01388500

61. $CH_3PH_2\cdots IBr$

0 1

P	-2.98576000	-0.43196600	0.00004400
H	-3.57225700	-1.14432900	-1.07157600
H	-3.57174200	-1.14398400	1.07217200
C	-3.93387700	1.15125000	0.00000100
H	-5.01118700	0.98277300	0.00015400
H	-3.65473800	1.72721200	0.88323900
H	-3.65496400	1.72704100	-0.88342300
Br	2.66912500	0.17439000	0.00003400
I	-0.10499700	-0.16378100	-0.00004600

62. $OHPH_2\cdots IBr$

0 1

H	3.66861700	-1.03646400	-1.06842700
P	3.02594400	-0.36503900	0.00002800
O	3.82397900	1.08014900	0.00003900
H	3.22524600	1.83549700	-0.00024100
H	3.66834400	-1.03627100	1.06876200
Br	-2.64977000	0.14920800	0.00002600
I	0.11695800	-0.15378600	-0.00003300

63. $CF_3PH_2\cdots IBr$

0 1

P	-2.18950400	-0.96977700	0.00032800
H	-2.70861400	-1.74685800	-1.06227500
H	-2.70571700	-1.74335700	1.06687600
C	-3.57028900	0.32956900	0.00005300
F	-3.46645300	1.11460200	1.08828200
F	-3.46912400	1.11123900	-1.09088700
F	-4.80672000	-0.20697000	0.00238500
Br	3.49359900	0.29834300	0.00024900
I	0.81289300	-0.23683800	-0.00031200

64. $CNPH_2\cdots IBr$

0 1

H	-3.44428000	-1.49607700	1.06551800
P	-2.90852300	-0.73415400	-0.00012600
H	-3.44379800	-1.49556500	-1.06637700
C	-4.12935300	0.56636100	-0.00006700
N	-4.83745400	1.47966000	-0.00000100
I	0.16662900	-0.14741100	0.00013100
Br	2.86636800	0.23031300	-0.00010800

65. $NO_2PH_2\cdots IBr$

0 1

H	-2.85707500	1.05904600	1.58620000
P	-2.48014000	-0.00317200	0.73263100
H	-2.85761000	-1.07210900	1.57753200
N	-4.07705700	0.00128700	-0.26899700
O	-4.51964000	1.09063600	-0.57975200
O	-4.52014300	-1.08529400	-0.58861000
Br	3.27149300	0.00068000	-0.12623700
I	0.55230600	-0.00028100	0.02820700

66. $CH_3PH_2\cdots F_2$

no complex

67. $OHPH_2\cdots F_2$

no complex

68. $CF_3PH_2\cdots F_2$

no complex

69. $CNPH_2\cdots F_2$

no complex

70. $NO_2PH_2\cdots F_2$

no complex

71. $CH_3PH_2\cdots FCl$

no complex

72. $OHPH_2\cdots FCl$

0 1

F	1.36313600	0.72229400	0.00028700
P	-2.74910700	0.22955400	-0.00026300
O	-1.32495400	-0.66797600	0.00051300
H	-3.43614300	-0.48010200	1.02837900
H	-3.43571500	-0.48122600	-1.02841600
H	-0.55842700	-0.08464500	0.00034900
Cl	2.76460500	-0.20906900	-0.00018000

73. $CF_3PH_2\cdots FCl$

0 1

P	0.07950800	-0.27722200	-0.00036000
C	1.98037900	0.04966300	-0.00002300
H	0.04239400	-1.14589800	1.10248700
H	0.04379800	-1.15405400	-1.09684500
F	-1.64021400	0.27512700	-0.00176600
F	2.33433000	0.74202700	-1.09153700
F	2.33162200	0.75033800	1.08716800
F	2.67824500	-1.10582100	0.00520800
Cl	-3.79393700	0.01207500	0.00048500

74. $CNPH_2\cdots FCl$

0 1

P	0.00000000	1.36645700	0.00000000
H	0.89066600	0.99530500	1.04202800
H	0.89066600	0.99530500	-1.04202800
F	-0.49423100	-1.62819000	0.00000000
C	0.48361200	3.09909100	0.00000000
N	0.67260500	4.23982300	0.00000000
Cl	-0.29077400	-3.30041500	0.00000000

75. $NO_2PH_2\cdots FCl$

0 1

P	-0.75176500	-0.00311900	-0.20665800
N	-2.61701500	0.00098700	0.05586900
H	-0.54392200	-1.04863200	0.72692100
H	-0.53900000	1.04213000	0.72606100
O	-3.16887900	1.08837600	0.09150400
O	-3.17356100	-1.08398400	0.09273300
F	2.07688100	-0.00024500	-0.34159300
Cl	3.68977100	0.00079100	0.16801500

76. $CH_3PH_2\cdots FBr$

0 1

P	2.45218200	-0.61391900	0.00000400
H	3.31319700	-1.03667400	1.05086500
H	3.31157800	-1.03631000	-1.05231800
C	2.83352300	1.20539300	0.00000300
H	3.90273300	1.41959300	-0.00050300
H	2.37476600	1.65710700	-0.88100300
H	2.37562700	1.65695100	0.88153700
Br	-1.94800400	0.06986300	-0.00004600
F	-0.09795800	-0.34771700	0.00033000

77. $OHPH_2\cdots FBr$

0 1

H	4.10762700	0.60292700	-1.06785500
P	3.47880000	-0.23596500	-0.10068300
O	2.11633500	0.70784700	0.18431200
H	1.31977400	0.16449100	0.17639300
H	4.30026900	0.26719800	0.95140600
Br	-2.12469700	0.08926600	-0.03833600
F	-0.49732600	-0.69802500	0.14639700

78. $CF_3PH_2\cdots FBr$

0 1

P	1.35587100	-0.43524400	0.08978800
H	1.04247300	0.18221500	-1.14958100
H	1.01965400	0.70639200	0.86343000
C	3.18812800	0.04219500	-0.01495900
Br	-3.57313900	0.00839500	-0.02500600
F	3.79896800	-0.17513700	1.17358400
F	3.82063300	-0.72214600	-0.93639200
F	3.42895100	1.33166600	-0.34679400
F	-1.76734000	0.13151200	0.09896700

79. $CNPH_2\cdots FBr$

0 1

H	1.88421600	0.65238900	1.04434400
P	2.20107200	-0.25517100	-0.00050300
H	1.88371800	0.65810500	-1.04021100
C	3.96012000	0.12747000	0.00015900
N	5.11005800	0.24919200	0.00024900
Br	-2.54788300	0.09738100	0.00006200
F	-0.79325000	-0.37782700	-0.00016400

80. $NO_2PH_2\cdots FBr$

0 1

H	-1.32590500	1.04882400	0.67642300
P	-1.56139500	0.00447600	-0.25107300
H	-1.32004300	-1.04318600	0.67112600
N	-3.41840700	-0.00149300	0.07482800
O	-3.97476700	1.08313400	0.13062700
O	-3.96902300	-1.08906100	0.12839800
Br	2.93965600	-0.00062000	0.09983300
F	1.18422900	0.00075300	-0.40795500
